# Gas-Phase Dynamics of Bundle Formation from High-Aspect-Ratio Carbon Nanotubes

**DOI:** 10.1021/acs.langmuir.4c02260

**Published:** 2024-09-30

**Authors:** Rulan Qiao, Xiaoyu Qiu, Adam Boies

**Affiliations:** † Department of Engineering, University of Cambridge, Cambridge CB2 1PZ, United Kingdom; ‡ Department of Mechanical Engineering, Stanford University, Stanford, California 94305, United States

## Abstract

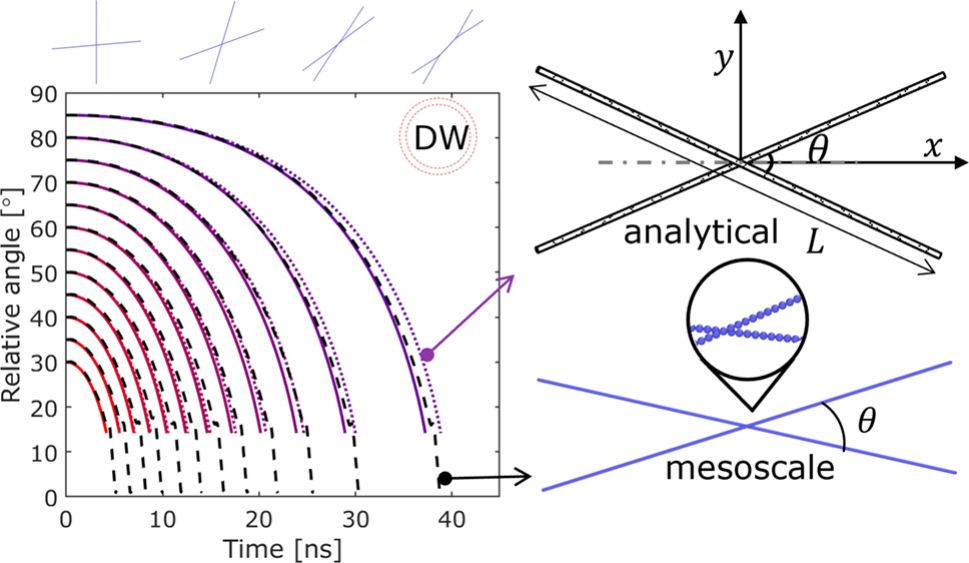

In floating catalyst chemical vapor deposition (FCCVD), high-aspect-ratio carbon nanotubes (CNTs) are produced in the gas phase at high number concentrations and undergo collision and agglomeration, eventually giving rise to a macroscale aerogel, enabling functional material forms such as fibers or mats to be obtained directly from the synthesis process. The self-assembly behavior between high-aspect-ratio CNTs dictates the resulting morphology at the nanoscale and subsequently the bulk properties of the CNT product. Reorientation between CNTs after collision is a critical step that results in bundle formation and precedes aerogel formation. However, it has been challenging to study the phenomenon with existing methods as it spans multiple time and length scales. In this study, a physics-based semi-analytical model was developed to study the gas-phase reorientation dynamics of high-aspect-ratio CNTs and their bundles, with ±10% accuracy compared with mesoscale molecular dynamics simulations, but at <0.1% the computational cost. It was revealed that the reorientation time scale is dictated by the interplay among the van der Waals potential, drag, and the geometric configuration of CNTs upon collision. This then allows the time scale of reorientation (i.e., bundle formation) to be compared with other gas-phase dynamics in a typical FCCVD reactor and offers new insights into the self-assembly behavior of 1D nanoparticles in the gas phase.

## Introduction

Carbon nanotubes (CNTs), in their various bulk material forms, have demonstrated great potential in a myriad of applications.^1^ The rising demand for better-performing materials in the aerospace, energy storage, and electrical sectors has opened broad markets for advanced CNTs, valued at $0.8 billion with 20% annual growth.^2^ Among the various synthesis routes for CNTs, the floating catalyst chemical vapor deposition (FCCVD)^3^ process is of particular interest for both the research and industry communities as it produces high-aspect-ratio CNTs that are typically longer than 10 μm, which gives rise to a macroscale aerogel in a single, continuous process, making it a scalable synthesis route with great potential for process intensification while offering a portfolio of different material forms that can be readily integrated into various applications.^4−6^

**Figure 1:**
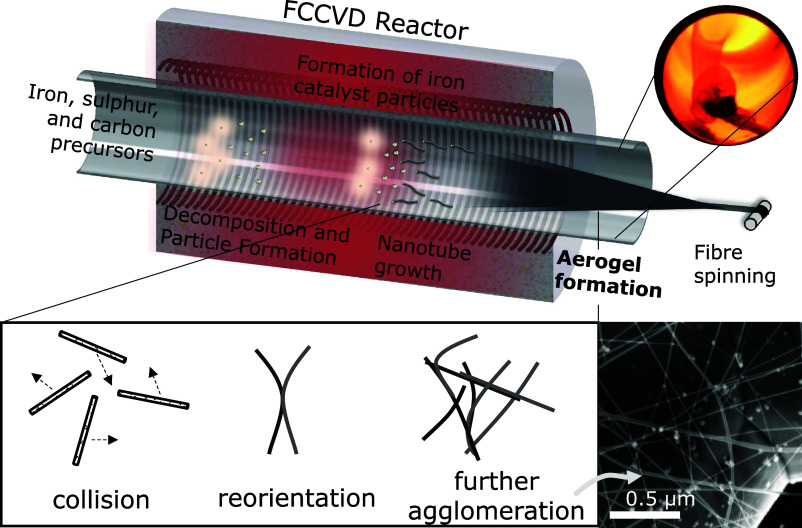
Schematic of the FCCVD reactor showing precursor delivery, catalyst particle formation, CNT growth, aerogel formation due to the agglomeration of CNT particles including both collision and reorientation, and the final fiber spinning process. The inset in the top right is a camera image taken from the downstream end of the reactor tube. The SEM image shows the network formed by CNT particles in the aerogel formation region.

In a typical FCCVD reactor as shown in Figure 1, carbon and catalyst precursors are delivered and decompose in the gas phase at high temperatures (>1000 °C). This is followed by the nucleation of catalyst nanoparticles and the subsequent catalytic growth of CNTs. Agglomeration between CNTs gives rise to a self-assembled CNT aerogel, which can then be “spun” into a fiber or collected as a 2D mat.^4,7^ The emergence of the self-assembled material is a key milestone as it marks the transition of the material from the nanoscale to the macroscale and subsequently to various bulk forms with useful functions in various applications. Understanding this self-assembly behavior offers opportunities to exert greater control over the synthesis process and product properties. For example, conductive transparent films made of networks of single-walled carbon nanotubes (SWCNTs) with less bundling showed a lower sheet resistance,^8^ whereas fibers consisting of CNTs with a higher aspect ratio or a higher degree of alignment exhibited higher conductivity and tensile strength.^9,10^ A fundamental understanding is beneficial not only for the FCCVD synthesis of CNTs but also for other 1D nanomaterials following similar synthesis chemistries, such as nanowires made of silicon and silicon carbide.^11,12^ Recent research progress has shone a light on catalyst dynamics,^13,14^ the effects of carbon precursors,^8,15^ and reactor designs,^16,17^ but due to the complex nature of the process, a clear understanding of the emergence of aerogel remains elusive. The self-assembly behavior of CNTs following collision in the gas phase dictates the resulting network structure at the nanoscale and subsequently the properties of the bulk product. Percolation theory could be useful in studying properties such as the electrical conductivity of CNT networks,^18−20^ but its static nature means it cannot be directly applied to study the kinetics of aerogel formation and the preceding dynamic processes.

Aerogel commonly refers to a high-porosity material consisting of solid colloidal or polymeric networks formed by a top-down method by drying a liquid gel.^21^ However, the formation of a self-assembled aerogel from aerosol systems, i.e., a bottom-up route, is a fundamentally different process governed by the agglomeration of aerosol particles, as shown in the schematic in Figure 1. The gas-phase phenomenon of aerogel formation was first observed and reported by Sorensen et al. in 1998 on ramified soot aggregates from an acetylene diffusion flame.^22^ Experimental studies to engineer the phenomena for the synthesis of other materials followed. Silica^23^ and carbon aerogel^24^ were produced by controlled detonation, and TiO_2_ aerogel was synthesized from a down-fired, buoyancy-opposed diffusion flame system.^25^ This was accompanied by theoretical investigations via various modeling techniques,^26,27^ which demonstrated the tendency of particulate systems to form a gel, where the particles do not coalesce after collision, but instead become ramified aggregates. However, the theoretical framework cannot be applied directly to the emergence of aerogel from 1D nanoparticles such as CNTs either, for they are known to form bundles upon collision,^28^ analogous to the coalescence of the spherical particles. If these 1D particles continued to coalesce indefinitely, forming an ever larger bundle, the final material would have been a single dense bundle of CNTs. However, the FCCVD material emerges as a self-assembled, low-density aerogel. This hence presents a suite of interesting questions: for gas-phase systems consisting of 1D nanoparticles, how does an aerogel form? When and how does the system transit from particle collision to network formation, i.e., the onset of gelation? What are the key parameters that govern whether the system will result in an aerogel? These questions motivated a series of studies. Boies et al. presented the first study of collision rates for 1D nanomaterials undergoing thermal transport via Langevin dynamics simulations, through which they calculated the time scales for CNT collision.^29^ The simulations also consider the probability distributions of the collision positions and collision angles. Kateris et al. then studied the time scale of CNT bundling with an adaptive mesoscale model that represented CNTs as a chain of nodes with intermolecular interactions described by a mesoscopic formulation of the Lennard-Jones potential and showed that the bundling time scale (τ_B_ ≈ 1 ns–1 μs for a CNT length of 0.1–10 μm) is much smaller than the characteristic collision time scale (≈0.1–10 s),^28^ resulting in immediate coalescence upon collision and corroborated the experimental observations of FCCVD samples where CNTs exist in bundles consisting of up to 20 individual CNTs. It is anticipated that as the CNTs increase in length and their bundles increase in size, subsequent reorientation may slow down such that the reorientation time scale approaches that of collision, prompting the formation of aggregate-like structures that are no longer one-dimensional.

The CNT modeling community has made tremendous progress in various fields over the past three decades from both the atomistic and the continuum perspectives.^30^ Due to the diversity in length scales, geometries, and target applications, the model is usually tailored for a specific application or for better understanding the relationship between molecular structures and a specific bulk property (e.g., electrical, thermal, or mechanical performance). The dynamics of CNT aerogel formation is an area that has not seen many in-depth studies. As the aerogel and the constituent high-aspect-ratio 1D particles span across several length scales and the kinetics of their formation across time scales that are orders of magnitude apart, it presents a significant modeling challenge. Mesoscale models, also commonly referred to as “bead–spring” models, reduce some of the atomistic degrees of freedom by representing the CNT molecule as a chain of beads that interacted with one another via spring-like multibody intermolecular potentials. The model was pioneered by Buehler^31^ and adapted over the years.^28,32,33^ It is particularly useful for describing assemblies of CNTs with high aspect ratios and several micrometers in length. Nevertheless, this bead–spring model cannot predict the dynamics of larger bundles due to the infeasible texture induced by the spherical interaction potential of the beads.^34^ A corrected and hence more accurate mesoscopic potential was then proposed by Zhigilei and Volkov with a mesoscopic force field method.^32,35,36^ The CNTs are represented by a series of cylindrical segments whose tubular potential is determined by integrating the continuum van der Waals (vdW) forces over all interacting segments. As a result, the formation of large networks, including bending buckling, can be correctly captured.^33^ However, the computational cost of such models increases rapidly as the CNT length extends beyond a few μm. When expanding the parameter space to include scenarios with longer CNT lengths, a greater number of walls, and larger bundles, even the adaptive mesoscale model^28^ would incur significant and infeasible computational costs. Therefore, new models with reasonable computational costs are needed to describe and understand the gas-phase dynamics of CNTs from FCCVD processes.

In this study, we advanced our analysis to meet the multiscale modeling challenge with first a mesoscale molecular dynamics (MD) model and then a semi-analytical model by approximating CNTs undergoing reorientation as rotating cylinders. The mesoscale model is capable of predicting the reorientation time for CNTs of various types at 0.1% of the time cost compared to that of the atomistic MD models. This allowed simulations over longer time scales (hundreds of ns) to be carried out for MWCNTs and bundles, which are impossible for atomistic MD models (a few ns). However, the computational cost of the mesoscale model is still too high for describing longer CNTs (over tens of microns) and larger bundles, which are often observed in a typical FCCVD aerogel.^29^ The expanded time scale of the mesoscale model still falls short of the characteristic collision time scale (0.1–10 s) by at least 6 orders of magnitude. Hence, we propose a semi-analytical model to study the reorientation dynamics of CNTs by drawing upon the physical insights gained from the mesoscale studies, but with <1% the time cost. The semi-analytical model provides insights into how reorientation time scales with key parameters such as initial collision angle, CNT length, bundle size, etc. Conversely, like all simplified analytical models, it is subject to the validity of underlying assumptions, which are also discussed. This multiscale modeling capability enabled us to study the CNT aerogel formation in FCCVD reactors and can aid broader research on gas-phase synthesis 1D nanostructures in general.

## Methods

Bundle formation, or the reorientation of CNTs upon collision, is driven by the tendency of the system to minimize its interaction potential. This motion is damped by drag in the gas phase. Embedded in both the analytical and mesoscale models in this study is the assumption that the nanotubes maintain a cylindrical shape. We based this assumption on the observation from atomistic simulations performed on two bundling CNTs^28^ as well as theoretical calculations where the thermodynamic persistence lengths of CNT and bundles were found to be in the macroscopic range (up to 1 mm for individual CNTs and even longer for bundles), as reported by Yakobson and Couchman.^37^ They also suggested that the entangled network structure is in a frozen, nonequilibrium state. In other words, CNTs with lengths up to 100 μm are unlikely to exhibit significant curvature in their shape if they are under only the influence of thermodynamic forces. Also assumed in both models is that surface deformation is negligible, such that the cross sections of CNTs remain circular. In practice, vdW forces may cause local “flattening” on the cylindrical surface, resulting in a stronger interaction between two CNTs. An extreme example is the formation of “dog-bone” cross sections where few-walled, large-diameter nanotubes collapse,^38−40^ which would increase the contact area between CNTs and maximize packing in the resulting bundles. He et al. found that the threshold diameter is about 5.1 nm for an SWCNT, a size that is unusually large for SWCNTs from typical synthesis conditions^40^ but possible if the size and composition of the catalyst nanoparticles are carefully controlled. FCCVD processes predominantly produce multiwall CNTs (MWCNTs) but can be tuned to produce SWCNTs^6,8^ with a range of CNT diameters, which typically do not exert control over catalyst nanoparticle sizes so as to favor this growth mode. We also assumed that effects due to nonlinear elastic phenomena can be ignored, such as bending, buckling of CNTs,^33,41,42^ and the rippling mode during bending of large-diameter CNTs.^41,43,44^

### Analytical Model

**Figure 2:**
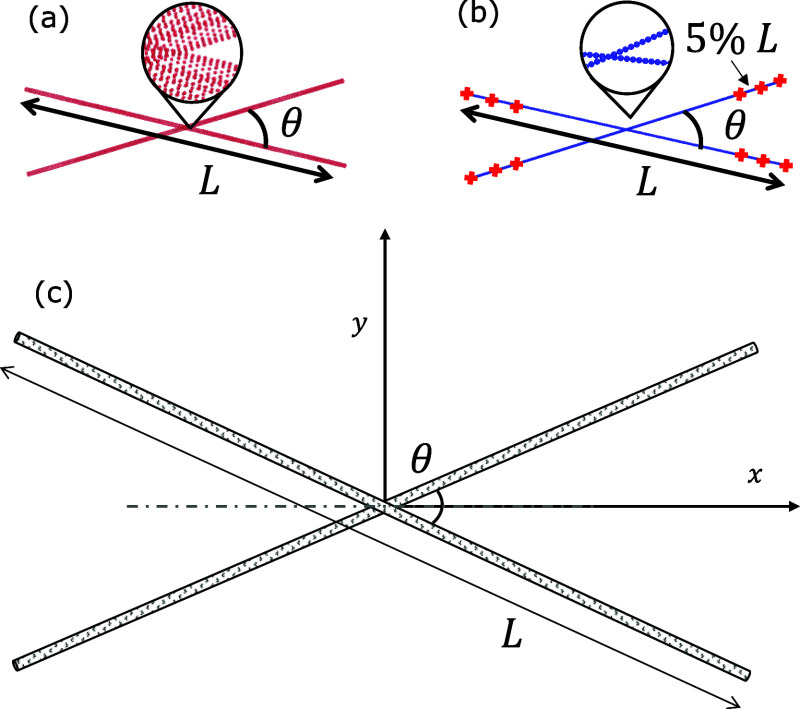
Schematic illustrating the configuration of two CNTs with length *L* and relative angle θ in (a) the atomistic model, (b) the mesoscale model, and (c) the analytical model. The red markers in (b) indicate the positions of the segments used for calculating the relative angle.

To gain insight into the dominating physical processes during the reorientation of CNTs, we examined the bounding behavior of the system. Treating the CNTs as rigid cylinders with the contact point at the center of both CNTs, the reorientation dynamics for one of the CNTs can be described by Newton’s second law for angular motion,
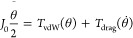
1where the driving force is due to the minimization of intermolecular potential and the motion is dampened by drag. Here, *J*_0_ is the second moment of inertia of a hollow tube and θ is the relative angle between the two CNT molecules, as shown in Figure 2c. Here, both CNTs are considered to rotate around the origin and toward the fixed, final bundling axis (*x*-axis in Figure 2c). For two CNTs of the same type, the final bundling axis coincides with the symmetry line (shown as a dotted line), and therefore, the angular position of the CNT lying in the first quadrant is θ/2. The torque due to intermolecular potential and drag are *T*_vdW_ and *T*_drag_, which are functions of relative angle θ and , respectively, as to be explained later.

#### Torque Due to Intermolecular Potential between Two CNTs

The torque due to vdW interaction, *T*_vdW_, was derived from the mesoscopic interaction potential between two infinitely long CNTs as a function of the relative angle θ and the shortest distance *h* between the axes of two straight CNTs by Volkov and Zhigilei. Volkov and Zhigilei showed that although the torque does differ slightly between two infinitely long CNTs and between CNTs of finite lengths, the deviation only starts to exceed 1% when the relative angle drops below 2° for two 100 nm (10,10) CNTs and that this threshold angle decreases with increasing length. For the typical range of CNT length *L* (hundreds of nanometers to tens of microns) and relative angle θ that are of interest in an aerogelation process, the simpler formulation shown in eq 2 based on two infinitely long CNTs is sufficient. It is also assumed that for MWCNTs, the mesoscopic interaction potential can be approximated by that of the most outer wall as the vdW interaction diminishes rapidly with distance.
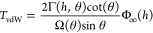
2where Volkov and Zhigilei defined the scaling functions as Γ(*h*, θ) = 1 + [Γ_⊥_(*h*) – 1] sin^2^(θ) and Ω(θ) = 1/(1 – *C*_Ω_sin^2^ θ). Φ_*∞*_ (*h*) is the integration result of the potential density function for parallel, infinitely long nanotubes at a certain *h*. *h* is the shortest distance between the axes of the CNTs. It was shown that for a given interatomic potential, there exists an equilibrium distance δ*h*_0_ between the outer surfaces of the two CNTs (δ*h*_0_ = *h*_0_ – 2*R*), at which the potential density is minimized.^32^ This equilibrium distance δ*h*_0_ is found to be 3.144 Å for two (10,10) CNTs and only increases very slightly with nanotube radius *R* (δ*h*_0_ = 3.151 Å for two (30,30) CNTs). Therefore, a constant value of δ*h* = 3.15 Å is used for the results presented in this study from the analytical model. Values of Γ_⊥_(*h*) can be calculated numerically following the method illustrated by Volkov and Zhigilei and via an implementation of the method in the publicly available C++ code cntpot.^45^ The value of *C*_Ω_ can be found numerically for various nanotube radii.^32^

#### Drag Due to Interaction with Gas Media

The torque due to drag, *T*_drag_, is derived from Dahneke’s formulation for drag on a cylinder in the free molecular regime, which considers the motion of nanosized particles in fluid flow.^46,47^ In free molecular flow, drag arises from the momentum transfer of collisions between the particle surface and the gas molecules.^47^ Dahneke considered rigid-body collisions for two types of momentum transfer (diffuse and specular scattering). Later, Liu et al. derived generalized expressions for drag on a cylinder in the free molecular regime with consideration for intermolecular interactions between the cylinder and the surrounding gas molecules and showed that Dahneke’s expression is a special case (i.e., rigid-body collision) that represents the lower limit. Here, we still adopt the simpler form from Dahneke, but as will be shown later, the model is set up such that the drag coefficient can be scaled. The expressions derived by Liu et al. are based on a linear combination of reduced collision integrals that account for the nonrigid-body effect and hence can be incorporated into the model so long as the values of the reduced collision integrals are available.

For a slender body of radius *R* and length *L* (2*R* ≪ *L*) without dynamic rotational effects, the drag force according to Dahneke’s formulation can be expressed as^46^, where *m*_g_ is the molecular mass of the gas, *k* is the Boltzmann constant, *T* is the temperature, *N* is the gas number density, *V* is the relative velocity of the body to the fluid, and φ is the momentum accommodation factor (0 ≤ φ ≤ 1). The torque due to drag is hence  (see the Supporting Information for a detailed derivation). The moment of inertia, , treats the CNT molecule as a long cylinder rotating around its center point, where the linear density ρ_*l*_ of the nanotube is calculated based on the radius of the CNT specified and the number density of carbon atoms in a graphene sheet. Therefore, the ordinary differential equation in eq 1 can be formulated as
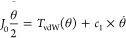
3

The initial conditions are set to be the initial relative angle upon collision, i.e., θ|_*t*=0_ = θ_0_, and zero initial angular velocity, i.e., |_*t*=0_ = 0, assuming zero rotational energy upon collision of the two CNTs. This latter assumption will be revisited later in the Results section. The stopping condition is when the relative angle has reached a predetermined critical angle θ = θ_c_, as introduced in detail in the next section. The equation is then solved with the ode45 solver in MATLAB 2020a.

#### “Off-Center” Collision and CNT Bundles of Dissimilar Lengths

Mesoscale simulations performed by Kateris et al. showed that there was no significant difference in the reorientation between two CNTs colliding “off-center” compared to those colliding at their center of masses, except for the cases where the contact point is at the end of the CNT (a rather rare event). Further, for two CNTs of dissimilar lengths *L*_1_ and *L*_2_ (*L*_1_ < *L*_2_), the reorientation time is similar to that between two similar CNTs of the shorter length *L*_1_. Therefore, while the proposed analytical model is based on two identical CNT molecules or bundles colliding at the center of both “cylinders”, it represents the most classic configuration following a collision between two long-aspect-ratio cylinders and accounts for the most important parameters that influence the reorientation dynamics. The results hence still give meaningful estimates for other “nonideal” configurations whose geometry deviates from that shown in Figure 2.

### Generalization of the Model to Bundle–Bundle Reorientation

Experimental observations of FCCVD products revealed that bundles with a wide diameter range (3–15 nm for SWCNT bundles and 30–50 nm for MWCNT bundles)^7,48^ could be found in the aerogel following typical synthesis conditions, suggesting that nanotube–bundle collision and/or bundle–bundle collision followed by reorientation creates larger bundles consisting of more than two CNTs. Therefore, it is of interest to examine the interaction between bundles. The analytical model, eq 1, allows for easy adaption by adding scaling parameters *k*_1_, *k*_2_, and *k*_vdW_, which can be determined by fitting mesoscale simulations This gives the following equation based on angular momentum
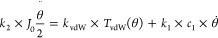
4

**Figure 3:**
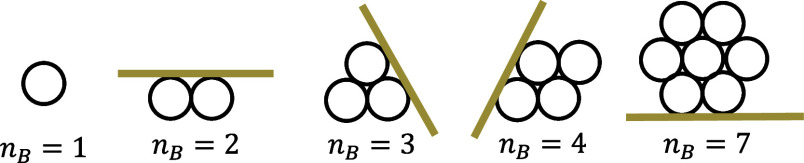
Cross-sectional views of possible layouts of CNTs (circles) in a bundle of size *n*_B_ and the interaction plane (solid lines).

We start by considering a simple scenario where a bundle of two CNTs (*n*_B_ = 2) interacts with another *n*_B_ = 2 bundle, assuming that the point of collision is again the center of the two bundles. By the principle of minimizing surface energy, so long as the two bundles have not collided with a relative angle of zero (forming a bundle of four straight away), four contact points are likely to form on the interaction plane, i.e., the plane on which nanotubes from the two bundles are in contact. Figure 3 shows the cross-sectional view of one bundle, where the solid line denotes the interaction plane where the contact points are to form. Thus, the analytical model proceeds to describe the reorientation motion following this collision by approximating each *n*_B_ = 2 bundle as a new cylinder. *k*_2_ accounts for the scaling of the second moment of inertia of the cylinder, and therefore shall be set to 2 for a bundle with *n*_B_ = 2; *k*_vdW_ accounts for the scaling of the vdW interaction and should be set to 4 because there are four pairs of contact between two *n*_B_ = 2 bundles.

If drag is included, then *k*_1_ shall be scaled by the increase in area in the plane that is normal to the direction of drag, which means it is still unity for *n*_B_ = 2, but shall be set to  for *n*_B_ = 3 and 4 and to  for *n*_B_ = 7. This simple adjustment does not account for sliding or twisting between CNTs within the bundle but treats the whole bundle as a new entity and assumes that the vdW interaction scales with the number of contact points in a bundle–bundle layout that minimizes surface energy upon collision.

### Estimation of the Critical Angle θ_c_

In earlier reports of mesoscale simulations, Kateris et al. identified two regimes during the reorientation of two CNTs, namely, the “central rotation” and the “zipping” regime.^28^ During “central rotation”, the relative angle θ decreases while no significant bending can be observed. As the reorientation progresses, sections near the contact point start to form a bundle, which then extends to the rest of the CNT in a “zipping” motion, accompanied by a rapid drop in intermolecular energy. Here, we define the angle at which bundling transitions from the former to the latter as critical angle θ_c_. θ_c_ marks the end of “central rotation” and hence is needed for the stopping condition of the analytical model. It is also an important parameter in the overall reorientation dynamics. It has been shown that for CNTs with large initial θ, the reorientation time is dominated by that spent in “central rotation”, but CNTs of different lengths would enter the “zipping” regime at a different relative angle.^28^ Therefore, it is important to have a reasonable estimation of θ_c_ in order to limit the analytical model to within its valid region. For CNTs or bundles with large initial θ, but small θ_c_, the reorientation will be dominated by time spent in “central rotation”, and hence, the time predicted by the proposed analytical model is representative of the overall reorientation time scale. However, for CNTs/bundles with a large θ_c_, it is more likely that the initial θ is close to or even smaller than θ_c_ such that reorientation may not undergo the “central rotation” phase at all but start directly in the “zipping” regime.

Here, we approach the problem with a simplified method with inspiration from Griffith’s theory on crack propagation,^49^ in that the transition from central rotation to zipping is viewed as the reverse process of the onset of crack propagation and fulfils the criterion of d*U*_tot_/d*t* = 0. We define the total energy *U*_tot_ of a system containing two CNTs as *U*_tot_ = *U*_vdW_ + *U*_bending_ + *E*_kinetic_. Here, *U*_vdW_ is the interaction potential between two CNT molecules due to vdW forces and can be expressed based on the results by Volkov and Zhigilei; *U*_bending_ is the strain energy stored in the molecules due to bending; and *E*_kinetic_ is the kinetic energy due to rotation. If one assumes the rate of change in kinetic energy is negligible at the onset of zipping, the criterion of d*U*_tot_/d*t* = 0 becomes
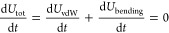
5

Here, we approximate the strain energy due to bending as , assuming simple bending with constant moment *M*, an effective total length of *L′*, and *M* ≈ *T*_vdW_ (θ) ≈ cot θ × *U*_vdW_ (θ).^32^ The bending force constant *k*_bending_ is calculated from the power-law fitted expressions proposed by Zhigilei et al. based on measurements from a series of atomistic MD simulations, as explained in the next section. By comparing with mesoscale results, *L′* is found to be half of the total length of the CNT. For MWCNTs, we approximated the bending force constant as the sum of *k*_bending_ of individual nanotubes that it comprises. Rearrangement of eq 5 would give , a numerical solution of which can be evaluated by a nonlinear solver. The estimated θ_c_ according to this method is compared to that observed in mesoscale simulations in Figure S3, showing an underprediction of ∼2° for all test cases by the analytical method. This discrepancy in the analytical model would lead to a 1–4% difference in the final results (i.e., time required for central rotation), which is not significant for the aim of this study. Therefore, θ_c_ estimated by this method is used as the stopping condition for describing the reorientation behavior between longer CNTs (*L* > 5 μm), which are common in FCCVD products, but for which mesoscale simulations would incur impractical computation costs. A maximum cutoff of 85° is set for θ_c_ because the method, based on the rate of change of intermolecular energy, would be inaccurate and not physically meaningful at such large relative angles. Since the critical angle decreases as the CNTs increase in bending stiffness (shown later in Figure 10), this cutoff threshold would only apply for extremely long and slender CNTs (e.g., DWCNTs with lengths greater than 50 μm as shown in Figure S3) and overall does not limit the applicability of the semi-analytical model.

### Mesoscale Simulations

Mesoscale simulations were used to validate the proposed analytical model following procedures detailed in a previous study on the aggregation of high-aspect-ratio SWCNTs.^28^ We extended the existing mesoscale model from SWCNTs to MWCNTs, with accurately defined input parameters, specifically, the stretching and bending force constants. The simulations were performed in LAMMPS^50^ with the mesoscopic potential determined via a mesoscopic force field method^32,35,36^ and implemented for LAMMPS by Philipp Kloza.^45^ The atomistic structure of the CNT is represented by mesoscopic CNT segments that are 1 nm long each, with both ends represented by particles as illustrated in Figure 2b. This model utilizes the Nóse–Hoover thermostat at 300 K and a time step of 1 fs. A longer time step results in numerical errors and abnormal simulation outcomes, while a shorter time step does not necessarily yield significantly improved results. The initial distance between adjacent CNTs is set to be 2 × *R* + 3.4 Å, where *R* is the equilibrium radius of the CNTs. The relative angle θ is calculated based on the position of the ends of CNTs in order to track the evolution of θ during the reorientation process, as illustrated in Figure 2b. The true relative angle between two CNTs at the contact point would be smaller if they are slightly bent under strong vdW forces. However, for simulating stiffer CNTs, such as MWCNTs and CNTs with higher chirality, they can be regarded as straight rods far from the contact point. Thus, the relative angle θ is evaluated as the average of three angles calculated from three sets of points close to the ends separated by 5% of the CNT length, as depicted in Figure 2b.

#### Estimation of Stretching and Bending Force Constants

In the mesoscale model of MWCNTs, both stretching, *k*_str_, and bending force constants, *k*_bnd_, are required as input parameters. However, *k*_str_ and *k*_bnd_ are merely available for SWCNTs from various computational methods, for example, tight-binding simulations and atomistic MD simulations^35,51−53^ Thus, we perform the first known atomistic MD simulations to evaluate *k*_str_ and *k*_bnd_ of MWCNTs.

We employed a dynamic method originating from the simple harmonic motion^54^ of CNTs when subjected to external forces. Simple harmonic motion is a periodic motion due to the restoring force exerted on an object when it is away from the equilibrium position. One of the typical simple harmonic oscillators is a weight vertically attached to a spring. The restoring elastic force from the spring makes the weight oscillate around the equilibrium position. At the position where the velocity of the weight is zero, all of the gravitational potential energy is transferred to the elastic energy stored in the spring, referred to as the “maximum position” in the following discussions.

**Figure 4:**
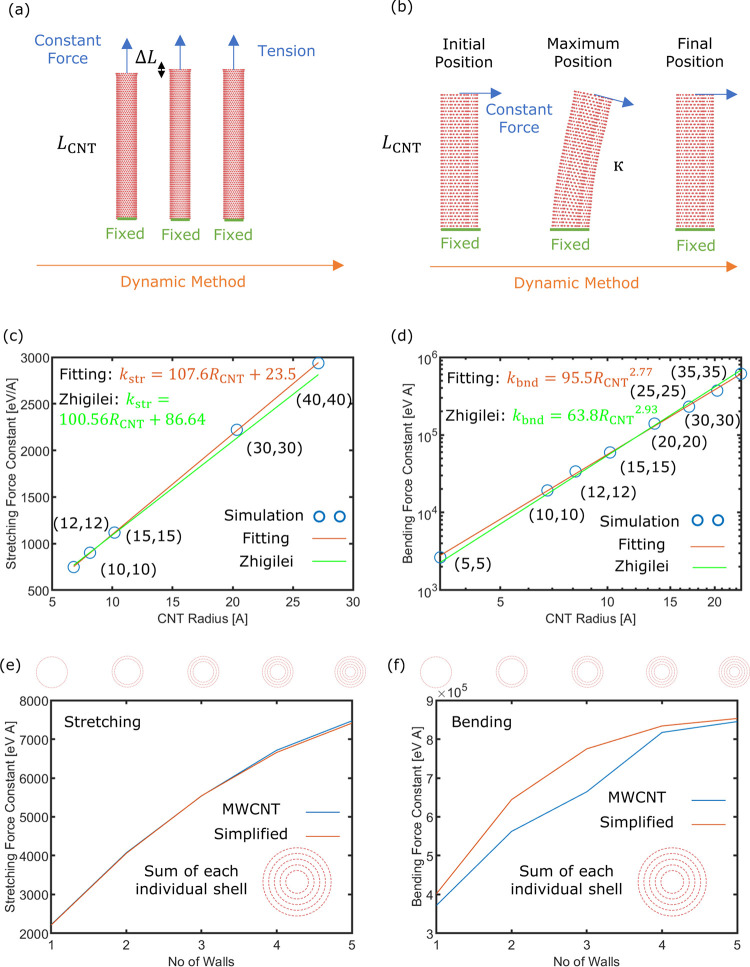
Schematic of dynamic simulations to calculate (a) stretching and (b) bending force constants. (c) Linear fitting of stretching force constants of nanotubes with different radii. (d) Power-law fitting of bending force constants of nanotubes with different radii. (e) Stretching and (f) bending force constants of (30,30) CNTs from SWCNTs to five-walled CNTs. Simplified force constants represent the mathematical outcomes attained by summing the force constants of each individual shell.

A similar scenario can also be observed while a CNT is stretched or bent by a constant force applied at one of its ends while the other end is fixed, as depicted in Figure 4a,b. Both ends are set to be rigid rings to avoid deformation due to the locally applied force. A periodic oscillation can be observed with the maximum position reached at the same time for a given CNT, even when the applied force varies. The constant force is equivalent to the gravitational force of the weight in a simple harmonic oscillator. The restoring force is the elastic force from the nanotube to minimize the increase in overall potential energy due to the stretching of carbon–carbon bonds while bending. At the maximum position, all of the work done by this constant force is transferred to the strain energy stored in the CNTs as depicted in Figure 4a,b. Thus, the energy is evaluated from the force, *F*_SHM_, and the displacement of *F*_SHM_, *l*_SHM_. The corresponding strain and curvature are also recorded at the maximum position. This periodic behavior persists until the applied force exceeds a critical threshold, causing the nanotube to buckle and thus undergo plastic deformation. Each simulation has a relatively short duration, typically spanning only a few picoseconds, which is much shorter than the conventional annealing simulations.^35^ This economical computational approach allows for the repetition of simulations on the same CNTs under various strains and curvatures. Thus, it is a feasible method to evaluate the force constant of MWCNT, which has significantly more atoms than the SWCNT.

In the atomistic models of CNTs, we adopt the widely used AIREBO potential for the interactions between all of the carbon atoms in LAMMPS.^55^ This AIREBO potential is composed of the short-ranged bond energy computed by the reactive empirical bond order (REBO) potential,^56^ the long-ranged vdW force described by the LJ potential, and the torsion energy if required. We repeat this dynamic simulation, with a corrected curvature, for SWCNTs of different chiralities to obtain an analogous relationship as Zhigilei et al. did. The corrected bending curvature, κ_corrected_, derived from integration is formulated as
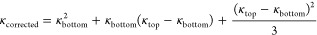
6where κ_bottom_ and κ_top_ are curvatures obtained from simulation results. The origin and derivation of this corrected curvature are detailed in the Supporting Information. The simulated bending and stretching force constants are shown in Figures S1 and S2.

The force constants obtained from our dynamic simulation exhibit a similar dependency on the radius of CNT, *R*, albeit with slightly different parameters as shown in Figure 4c,d. Zhigilei’s equations and fitted results from our dynamic method are plotted in green and red lines, respectively. The linear empirical stretching relationship in Figure 4c based on our simulation results is
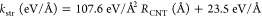
7The resulting bending relation (Figure 4d) taking the form of a power law is
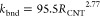
8Similar to the findings of Zhigilei et al., we also obtained the expressions for the radius-dependent force constants that align well with the simulation results. Thus, we adopt this dynamic method to calculate the force constants of the MWCNT, as required for the mesoscale model.

The stretching and bending simulations of (30,30) CNTs from single-walled to five-walled CNTs are plotted in Figure 4e,f, respectively. (30,30) MWCNTs refer to CNTs with a (30,30) CNT as their outermost shell, as illustrated in the top region of Figure 4e,f. Five-walled CNT has (30,30) and (10,10) as its outermost and innermost shell, respectively. The blue lines in Figure 4e,f are the force constants directly obtained from the dynamic simulations. The red lines represent the simple method of summing the force constants of each individual shell calculated by eqs 7 and 8. It can be observed that the simplified evaluation overestimated the bending force constants. The simulation results (blue) closely resemble (±7%) those obtained from the simple method (red), which only requires arithmetic calculation. The simplified model assumes that the bending stiffness is simply the sum of the bending stiffnesses of the individual SWCNTs comprising the MWCNT, without accounting for interactions between the walls. In our atomistic simulations, both sliding and flattening effects are captured through interlayer atomic interactions and the deformability of the CNTs. We observed that the nanotubes exhibit partial flattening during bending, which reduces the bending stiffness. On the other hand, shear interactions between walls could increase the bending stiffness. These two effects—flattening and interlayer shear—appear to partially cancel each other out, leading to the lower stiffness observed in the atomistic simulations compared to the simplified model. The influence of the interactions between walls is vanishingly small compared with the stretching of the carbon–carbon bonds. Thus, we adopt the empirical relations of force constants using Zhigilei’s relations fitted with our results for mesoscale simulations of MWCNTs in this study.

#### Validation of the Mesoscale Model against Atomistic Simulations

The mesoscale model for SWCNTs is validated and applied by Kateris et al. to investigate the bundling dynamics.^28^ However, MWCNTs are the primary CNT form from the FCCVD process. Thus, we parametrize the mesoscopic model in order to accurately simulate the dynamics of MWCNTs. We assume that the interactions between MWCNTs are equivalent to the interactions between their outermost shells, neglecting the influence of the inner shells and any local deformation for simplicity. We also make an assumption that the force constants of MWCNTs can be defined as the sum of the force constants of each individual shell (Figure 4e,f). This MWCNT mesoscale model is validated by comparison with an atomistic model as detailed in the Supporting Information (Figure S4), which showed good agreement after accounting for the decrease in reorientation time in the atomistic model due to flattening. Thus, we will adopt this optimized MWCNT mesoscale model in the following simulations.

## Results and Discussion

### Comparison between the Analytical and Mesoscale Models

#### Reorientation between Two CNTs at Rest

**Figure 5:**
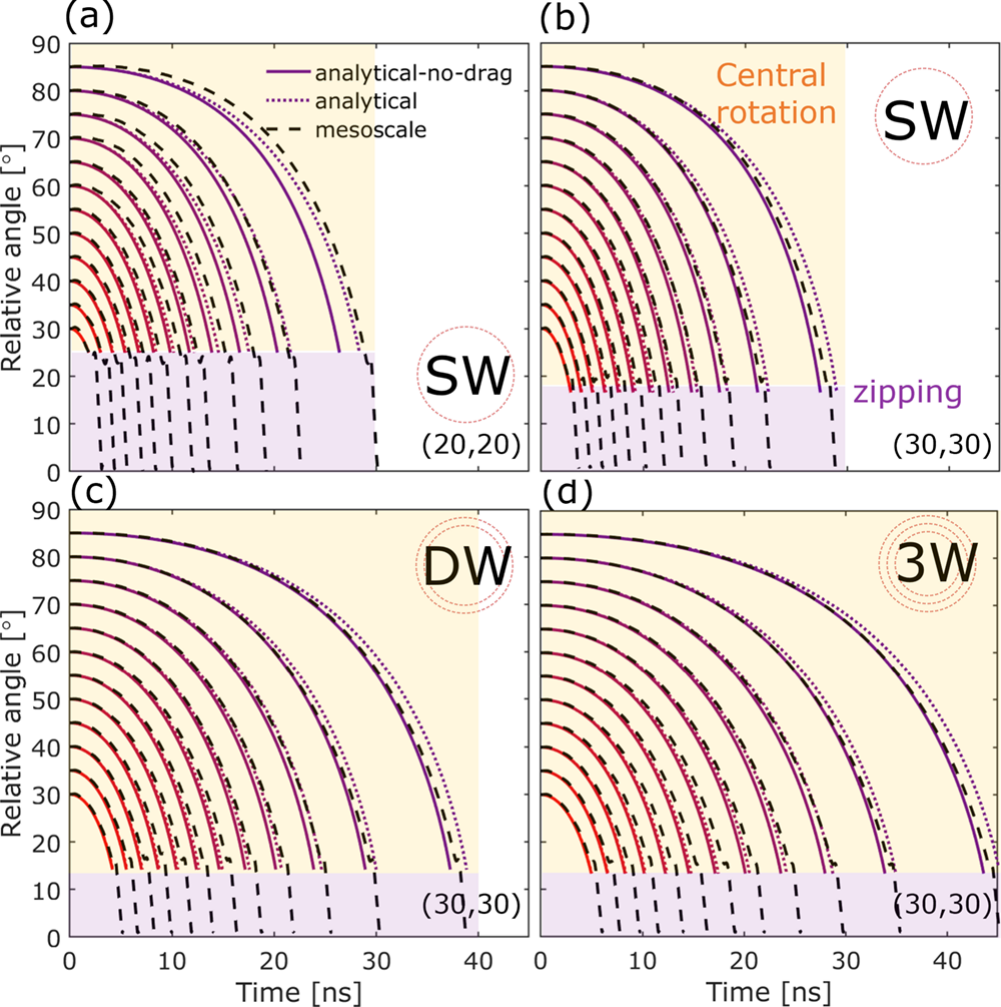
Mesoscale and analytical model predictions for the reorientation dynamics by tracking the relative angle θ with time for two 0.5 μm CNTs consisting of (a) single-walled (20,20), (b) single-walled (30,30), (c) double-walled (30,30) and (25,25), and (d) three-walled (30,30), (25,25), and (20,20) nanotubes.

**Figure 6:**
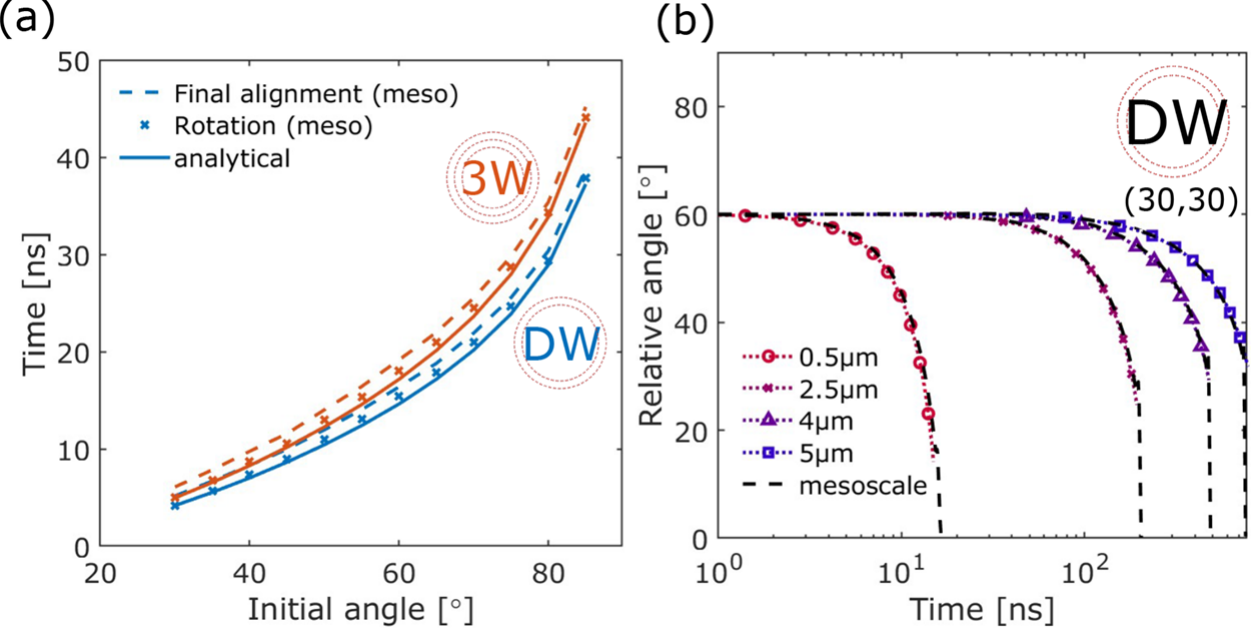
Mesoscale and analytical model predictions for (a) the reorientation time as a function of θ_0_ for CNT length *L* = 0.5 μm, and (b) the evolution of θ between two DWCNTs of various lengths (*L* = 0.5, 2.5, 4, and 5.0 μm). All DWCNTs have a (30,30) outer tube and a (25,25) inner tube.

The evolution of the relative angle θ is comparable between the analytical and mesoscale simulations. The reorientation proceeds slowly initially and then accelerates as θ drops, as shown for various CNTs with a length of 0.5 μm and an initial relative angle θ_0_ ranging from 30° to 85° in Figure 5a–d. This range of initial angles is chosen to focus on cases where the “central rotation” phase (highlighted in yellow in Figure 5b) is likely to dominate. Notably, the case with a precise initial angle of 90° with zero initial rotational energy is excluded as that configuration falls into a pseudoequilibrium state (discussed later). The dashed lines represent results obtained from the mesoscale simulations. Figure 6a shows that as the initial relative angle increases from 30° to 85°, the reorientation time progressively increases. This is dominated by the increase in time spent during the “central rotation” phase, as the time spent in the “zipping” phase is minimal in comparison. This “zipping” phase is characterized by a rapidly decreasing θ. Reorientation time also increases as a result of a larger nanotube size (see Figure 5a vs b), a greater number of walls (see Figure 5 b–d), or longer lengths (see Figure 6b). Due to the high computational cost for mesoscale simulations with a large domain size, for CNT lengths of 2.5, 4.0, and 5.0 μm, only the cases with θ_0_ = 60° were modeled for validation purposes.

Figure 5 shows two sets of analytical results, the “analytical-no-drag” (solid line) and the “analytical” (dotted line) against the mesoscale simulations (dashed line). The former has the drag term disabled (i.e., *k*_1_ = 0), while the latter analytic model includes the use of a scaled drag term with *k*_1_ = 0.2. As gas–CNT interactions are not accounted for in the mesoscale simulations due to the exceptionally high computation cost and the lack of calibrated mesoscale interaction potentials, it might appear reasonable to compare the mesoscale model with the analytical model that does not include drag either, i.e., “analytical-no-drag”. Indeed, the overall trend of θ during the reorientation process can be captured by the “no-drag” model, which slightly underestimates the reorientation time by 2–10% for the 0.5 μm long CNTs tested as shown in Figure 5. The discrepancy increases with the initial relative angle and decreases for CNTs with greater linear density and bending stiffness, for which the assumption of approximating CNTs as rigid cylinders is more valid. The discrepancy is small (2–10%) for the test cases shown in Figure 5 for 0.5 μm CNTs, but when the CNTs are 5 μm long, which is still within the typical length range for CNTs produced via the FCCVD,^7^ the “no-drag” analytical model would underestimate the reorientation time spent in central rotation by 45%.

Examining the setup of the mesoscale simulations more closely, although gas–CNT interactions have not been accounted for, due to the Nóse–Hoover thermostat used, the mesoscale model maintains the isothermal condition by effectively damping the system with a “numerical drag”. To achieve greater agreement between the analytical and mesoscale models, a suitable fitting parameter for the analytical model was found by comparing results against data from the mesoscale model for CNTs of various types (Figure 5) and lengths (Figure 6b) such that good agreement between the two models is achieved with *k*_1_ = 0.2. This parameter effectively corrects the discrepancy for all examined test cases and hence was included for all subsequent studies, unless otherwise specified.

#### Bundle–Bundle Reorientation

**Figure 7:**
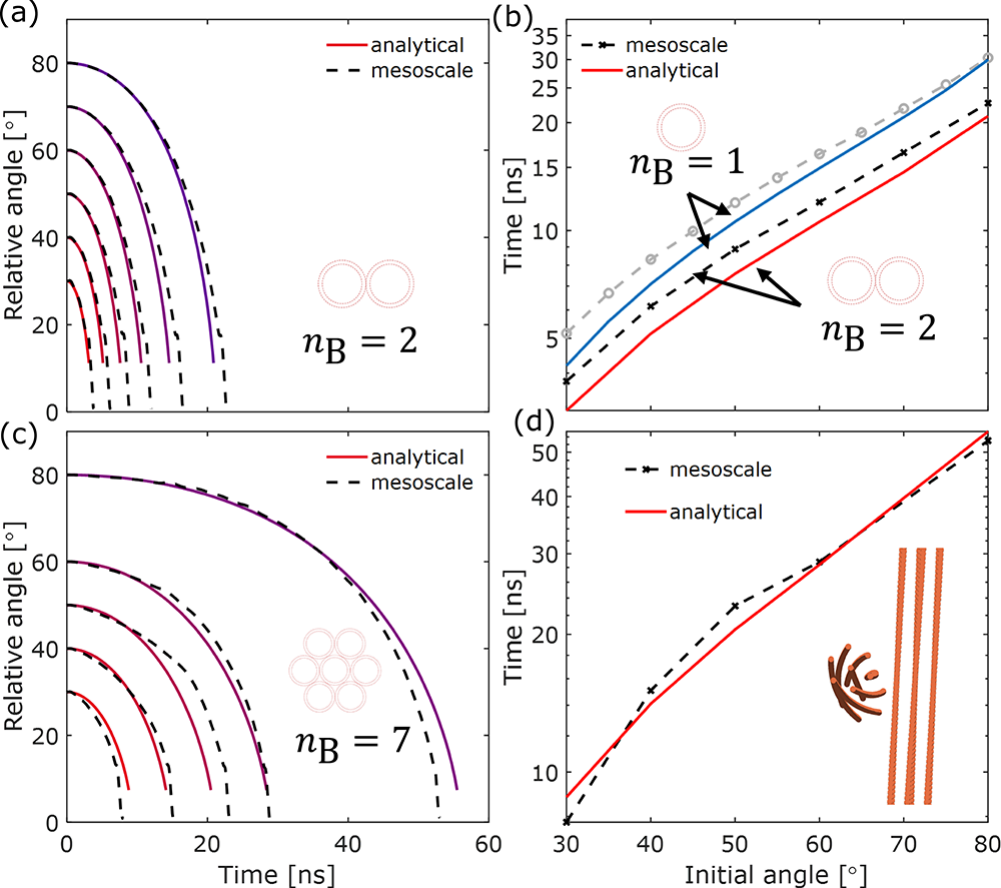
Reorientation between two (a,b) *n*_B_ = 2 bundles shown as a change in relative angle (θ) as a function of time. Each bundle consists of two 0.5 μm DWCNTs, with a (30,30) outer wall and a (25,25) inner wall. Drag has been scaled (i.e., *k*_1_ = 0.2) for the “analytical” case. Other scaling factors are *k*_2_ = 2 and *k*_vdW_ = 4. The same was shown for two *n*_B_ = 7 bundles (c,d) where scaling factors are *k*_2_ = 7 and *k*_vdW_ = 2.

To validate the analytical model for describing bundle–bundle reorientation, we compare results from the analytical and mesoscale simulations between two *n*_B_ = 2 bundles (Figure 7a,b) and two *n*_B_ = 7 bundles (Figure 7c,d), where the bundles consist of 0.5 μm DWCNTs. These two bundle sizes are selected because they represent two cases of particular interest. *n*_B_ = 2 bundles would be the dominating species after collision and reorientation between two individual CNTs, whereby a *n*_B_ = 7 bundle (diameter ∼ 12 nm) is the smallest unit with hexagonal packing, as shown previously in Figure 3. For a bundle size of *n*_B_ = 2, four contact points are expected to form, and hence, *k*_vdW_ is scaled to 4. Satisfactory agreement (<5% for central rotation, <10% for total alignment) between the analytical and mesoscale simulations is found. Interestingly, upon comparison of this set of results with those from the reorientation of two individual DWCNTs, reorientation is faster for *n*_B_ = 2 bundles than for individual CNTs (Figure 7b). This is because even though the moment of inertia has doubled, the interaction potential has increased more substantially (scaled by four times). As the bundle size increases, the moment of inertia scales linearly with *n*_B_, but the torque due to the vdW potential scales approximately with the number of contact points between the two rotating entities, causing the reorientation time to decrease for *n*_B_ = 2 and then increase for *n*_B_ > 2 (Figure S5).

Following from our hypothesis for scaling the analytical model for bundle–bundle reorientation, a *n*_B_ = 7 bundle should also have the vdW scaling at *k*_vdW_ = 4. However, on comparing with results from mesoscale simulations (Figure 7c,d), we found that an effective scaling factor of *k*_vdW_ = 2 yields a much better agreement (<10% discrepancy in reorientation time). This is likely due to twisting within the bundle, as shown in Figure 7d, such that instead of maintaining the four contact points within the rotation plane, two contact points are effectively lost as the internal twisting moves them out of the interaction plane. Due to the high computational costs of the mesoscale simulations for MWCNT bundles, data are not yet available to validate bundles with *n*_B_ = 3–6. However, the validation tests performed above can provide an estimation of the lower and upper bounds for the reorientation time of small bundles (*n*_B_ ≤ 7), which is of the utmost relevance to aerogel formation in an FCCVD process.

By converting the physical insights from mesoscale simulations into the scaling factors of the analytical model, one can make a reasonable prediction of reorientation time with a much reduced computational cost. This is critical if such models are to be used in continuum scale simulations that include fluid, energy, and chemical kinetics. The analytical model, combined with a suitable choice of scaling factors, is able to capture the dynamics of bundle–bundle realignment. The choice of scaling factors should reflect the configuration near the contact point of the two bundles. As the bundle size increases, phenomena such as internal twisting may significantly change the interaction plane between the two bundles, resulting in a weaker interaction and longer reorientation time. In other words, bundle–bundle reorientation dynamics is sensitive to the intermolecular interaction near the contact point. In a realistic reaction environment, there may be other factors that also affect the surface conditions near the contact point, e.g., surface contamination, trapping of catalyst nanoparticles that block the bundling pathway, carbon fiber growth on existing CNTs,^9^ etc.

### Reorientation Dynamics

#### Reorientation Time Increases with Length, Initial Collision Angle, and Linear Density

**Figure 8:**
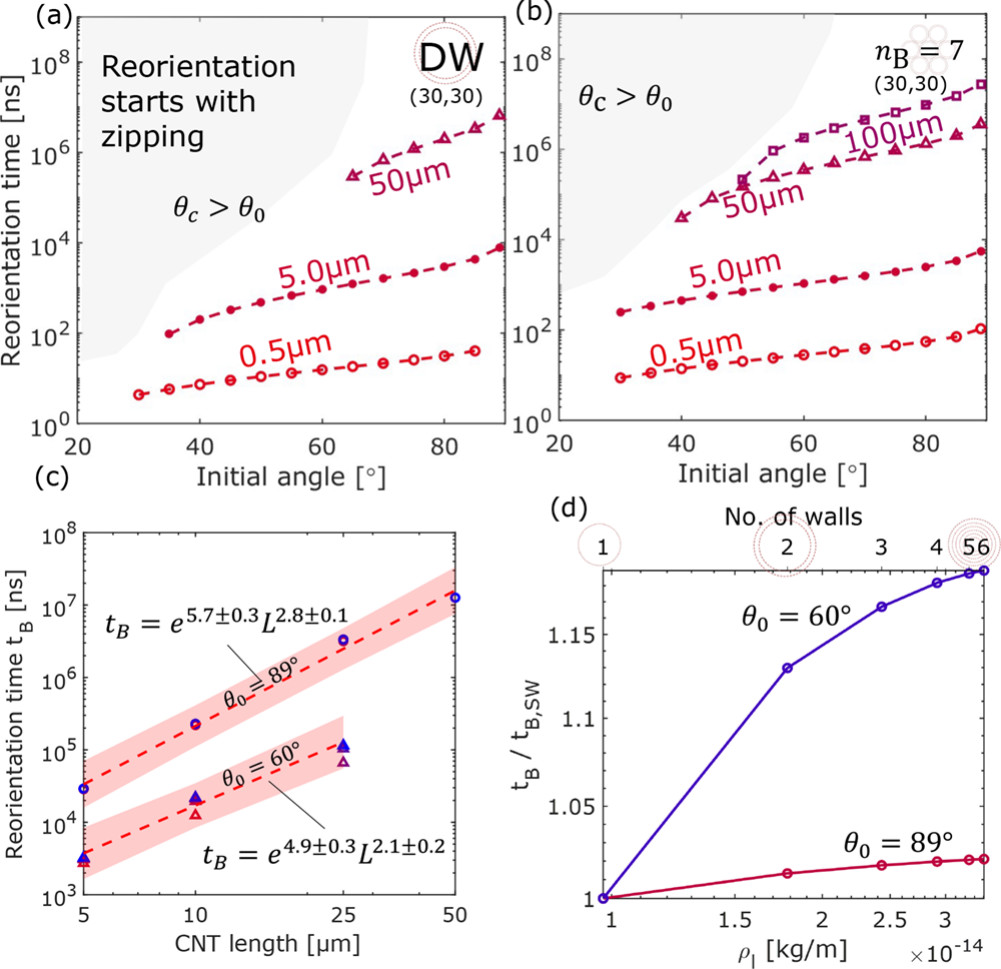
Reorientation time between (a) two DWCNTs and (b) two *n*_B_ = 7 bundles for various initial collision angles and lengths, where each bundle consists of seven DWCNTs, with a (30,30) outer wall and a (25,25) inner wall. (c) Reorientation time as a function of CNT length for θ_0_ = 60 and 89°. Highlighted regions represent 95% confidence interval for power-law fit. (d) Reorientation time as a function of the number of walls, normalized by that between two 0.5 μm long SWCNTs. Analytical simulations included a drag coefficient of *k*_1_ = 0.2.

With reasonable agreement found when comparing against mesoscale modeling results (±10% for reorientation time), the semi-analytical model is applied to explore how the reorientation time changes as a function of CNT length (*L*), initial collision angle (θ_0_), and the type of CNTs, to determine when the time scale of reorientation would match that of collision (indicative of the onset of aerogel formation). Results are shown in Figure 8a,b, where we studied the reorientation between CNTs or bundles with lengths up to *L* = 100 μm. Lee et al. found CNTs up to 50 μm in their final products, with an FCCVD reactor featuring a deep-injection delivery method for the precursors.^16^ Therefore, we intend to cover the lower and upper bounds of typical CNT lengths in our analysis. The range explored for the initial angle is from the critical angle up to 89° (Figure 8a,b), with the latter intentionally chosen to be just below 90° to avoid a pseudoequilibrium state (discussed further below). Shown in Figure 8a is the reorientation time *t*_B_ for two DWCNTs, where the reorientation time is plotted against the initial angle for different CNT lengths. As the CNT length increases, the critical angle also increases. Therefore, the regime where the reorientation would start with zipping directly is excluded (gray region) in Figure 8a,b. As observed for both tube–tube reorientation and bundle–bundle reorientation, a large initial collision angle (e.g., 89°) increases the reorientation time by 1–2 orders of magnitude compared to a smaller initial collision angle.

The dependency of *t*_B_ on the CNT length *L* is shown in Figure 8c for logarithmic scales of time. A power-law fit is found for the bundling time, where *t*_B_ = *e*^5.7±0.3^ · *L*^2.8±0.1^ for θ_0_ = 89° and *t*_B_ = *e*^4.9±0.3^ · *L*^2.1±0.2^ for θ_0_ = 60° (*L* in μm and *t*_b_ in ns). According to the Langevin simulations by Boies et al., which provide a statistical distribution of collision angles between two CNTs, θ_0_ follows a normal distribution with a mean of π/2 and a standard deviation of π/5. In other words, approximately 59% of the collision events would have a resulting reorientation time falling in the region bound by the data shown in Figure 8c.

The second moment of inertia, *J*_0_, of the nanotubes/bundles depends on the structural configurations, e.g., the number of walls per tube and the number of tubes per bundle. Figure 8d shows how reorientation time, normalized by that between two SWCNTs, changes with the number of walls as it increases from one to six, while the outermost shell remains to be a (30,30) nanotube. Unsurprisingly, there is a positive correlation as the increasing second moment of inertia slows down the reorientation, although the dependency is relatively weak. A 3-fold increase in linear density would result in up to a 3–20% increase in the reorientation time, depending on the initial angle.

In summary, the reorientation time spent on central rotation can be captured by a semi-analytical model with a range of conditions that, to the best of our knowledge, reasonably represent the CNTs and their bundles encountered in an FCCVD process. We concluded that the dominating factor causing an increase in reorientation time is length, followed by the initial collision angle θ_0_ and linear density ρ_l_. The reorientation time *t*_B_ follows a power-law relationship with the CNT length, where the fitted power index can range from 2.1 to 2.9 depending on the initial collision angle. An increase in θ_0_ could also result in a longer *t*_B_ by up to 2 orders of magnitude. An increase in linear density due to a greater number of walls would yield a greater *t*_B_ as well but to a lesser extent. A larger bundle size *n*_B_ does not always prolong the reorientation process as the behavior is also affected by the contact configuration between two bundles.

#### Effect of Initial Rotational Energy

**Figure 9:**
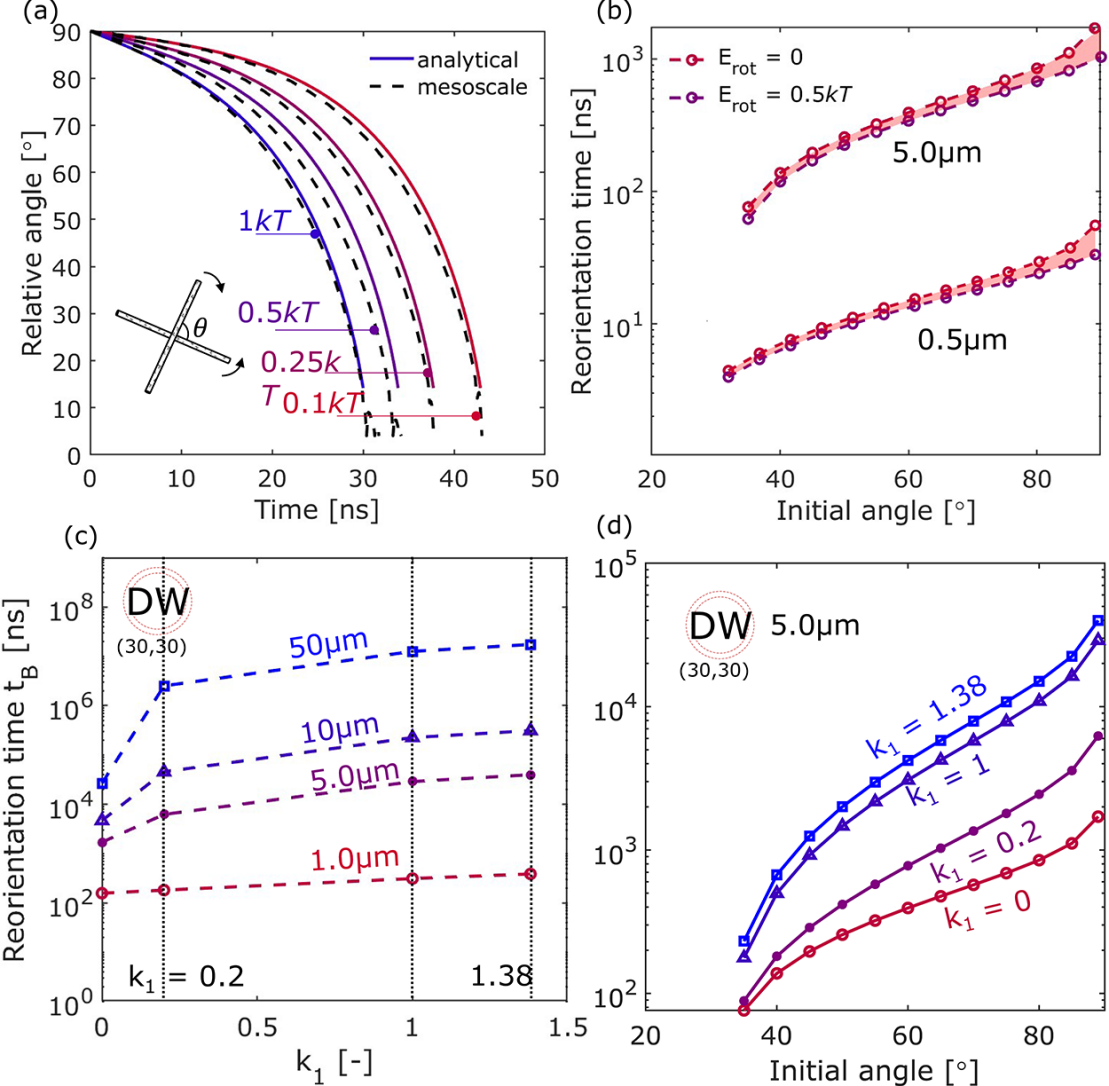
(a) Reorientation between two DWCNTs with (30,30) as the outer wall and (25,25) as the inner wall from an initial relative angle of 90° by simulations from the mesoscale and the analytical models. (b) Reorientation time with and without initial rotational energies for DWCNTs of 0.5 and 5 μm in length. (c) Effect of the scaling factor of drag, *k*_1_, on the reorientation time for two DWCNTs of various lengths and an initial collision angle of 89°. (d) Reorientation time between two 5 μm DWCNTs from various initial collision angles with different scaling factors of drag *k*_1_.

As shown earlier, the reorientation time is a strong function of the initial relative angle, and it can increase by an order of magnitude when θ_0_ increases from a value close to the critical angle to ∼85°. One can therefore expect that the reorientation time will keep increasing as θ_0_ approaches 90° but reaches a “pseudoequilibrium” state when the angle is exactly 90°, where both mesoscale and analytical simulations would predict no bundling behavior, but in reality, any perturbation would trigger the reorientation. In the absence of any external force, such a perturbation can still be caused by the rotational component of the inherent kinetic energy of the CNT molecule, *E*_rot_. Gas kinetic theory has shown that the average translational kinetic energy per molecule is *E*_kin_ = 3*kT*/2. Hence, a reasonable range for *E*_rot_ should be of the order ∼ *kT,* while the direction of rotation relative to the contact point is random. It is possible to investigate the effect of initial *E*_rot_ with the analytical model by replacing the zero initial condition of the angular velocity (θ̇ = 0) with an angular velocity based on *E*_rot_. Therefore, we investigate the effect of *E*_rot_ at the extreme case where θ_0_ = 90° and the two CNTs rotate within the bundling plane toward each other, i.e., the directions of rotation are opposite, as shown in Figure 9a. This modification is implemented in the analytical model by setting the initial conditions to be θ_0_ = 90° and . The reorientation behavior predicted by the analytical model is in general good agreement with those from the mesoscale simulations. Thus, the analytical model is used to predict the *t*_B_ for longer CNTs (5 μm), with and without an initial rotational energy with a magnitude of *kT*/2, as shown in Figure 9b.

When all other conditions are the same, the larger the initial relative angle, the more sensitive the bundling time is to the initial rotational energy, i.e., the reorientation behavior of two CNTs colliding at a relative angle of 90° is the most sensitive to small fluctuations in the initial rotational energy. This is because a larger fraction of the reorientation process would have been spent at a larger relative angle, during which *T*_vdW_ stays relatively small and constant, resulting in a slow acceleration. It is worth noting that despite the enhancement effect of an initial rotational energy in the “right” direction, the overall reorientation time remains in the same order of magnitude. In the case of two 5 μm long CNTs colliding at an initial angle of 85°,  of the initial rotational energy would result in approximately 27% reduction in the reorientation time, whereas it would result in 40% reduction for an initial angle of 89–90°

#### Effect of Drag

To investigate the effect of drag on the reorientation time, *k*_1_ was scaled for different CNT lengths, as shown in Figure 9c. As mentioned in the generalized form of the semi-analytical model (eq 4), the drag term can be disabled or scaled as needed. Later shown in the cases comparing analytical and mesoscale results (Figure 5), the drag term (i.e., *k*_1_ × *c*_1_ × θ̇) was scaled by setting *k*_1_ = 0.2 so as to match with the “numerical drag” caused by the isothermal condition used in the mesoscale simulations. Drag due to gas–CNT interaction cannot be characterized by the mesoscale simulations without significant sacrifice on computational cost. However, a suitable scaling factor should fall in the range of 1–1.38 for a CNT with an outer tube of the type (30,30). No scaling, i.e., *k*_1_ = 1, represents the case where the drag coefficient is derived based on the assumptions of rigid-body collision and a momentum accommodation factor of 0.9. Liu et al. showed that the nonrigid-body effect can be accounted for with a more universal formulation of the collision integral. Following their proposed model and the collision integral calculated for CNTs and nitrogen, the drag coefficient would see an enhancement factor of 1.38 when the outer radius of the CNT is around 2 nm, assuming φ = 0.9. The exact numerical value of this enhancement factor would increase with decreasing CNT radius and will be a function of the interaction potential between the CNT molecules and the gas molecules.

Incorporation of drag would result in a longer reorientation time, unsurprisingly. The effect becomes more prominent as the CNT lengthens (Figure 9c), as the first-order coefficient *c*_1_ depends on the CNT length to the power three. For example, when the CNT length is ∼50 μm, the reorientation time between the cases without and with drag can be increased by 2 orders of magnitude. When drag is included, i.e., *k*_1_ > 0, increasing the scaling factor *k*_1_ has the expected effect of varying the first-order coefficient in a second-order ordinary differential equation. Even though eq 4 does not have an analytical solution, the torque due to the vdW interaction *T*_vdW_ changes very slowly until the relative angle θ drops to a relatively low value (∼30°), which gives an approximate linear relationship between *k*_1_ and *t*_B_. Thus, mesoscopic simulations of CNT reorientation that ignore the interaction with gas molecules but use a thermostat that incurs a “numerical drag” (*k*_1_ = 0.2) can underestimate *t*_B_ by up to 85% (), whereas analytical simulations that ignore drag completely (*k*_1_ = 0) would underestimate *t*_B_ by orders of magnitude.

#### Effect of Length and Bending Stiffness on Critical Angle

**Figure 10:**
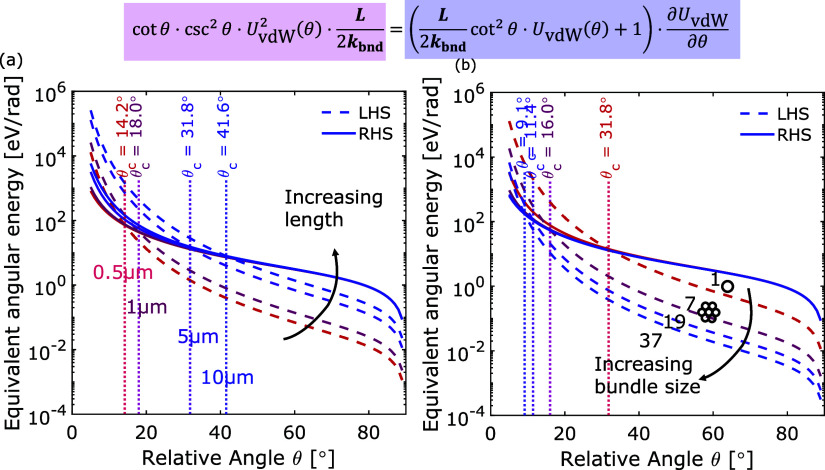
Estimation of the critical angle θ_c_ for (a) two DWCNTs with increasing length (0.5–10 μm) and for (b) two 5 μm long CNTs or bundles with various bundle sizes *n*_B_, whose overall bending stiffness *k*_bnd_ is approximated as the sum of the bending stiffness of the constituting CNTs.

Used as the stopping condition for the semi-analytical model, θ_c_ is an important parameter for estimating the time spent on the central rotation phase of reorientation. Therefore, it is of interest to understand the factors affecting the value of θ_c_ according to the proposed method explained earlier by examining the rearranged equation that matches the rate of change in bending and vdW potential energies (shown at the top of Figure 10).

The angular energy is shown as a function of relative angle in Figure 10 by plotting the LHS and RHS of the rearranged equation as a measure of the equivalent angular energy such that the intersection point denotes the critical angle. The critical angle θ_c_ strongly depends on the CNT length *L* and bending stiffness *k*_bnd_. As the CNT length *L* increases from 0.5 to 10 μm, θ_c_ increases from 14.2° to 41.6° for the reorientation between two DWCNTs. However, as the bundle size *n*_B_ increases from 1 to 7, θ_*c*_ would decrease from 32° to 16° due to the increase in bending stiffness *k*_bnd_. Although both parameters (i.e., *L* and *k*_bnd_) are present on both sides of the rearranged equation shown at the top of Figure 10, the LHS expression is directly proportional to *L*/*k*_bnd_, shifting the intersection point accordingly. Therefore, the value of the critical angle θ_c_ is larger for longer CNTs but smaller for larger bundles.

### Time-Scale Analysis

**Figure 11:**
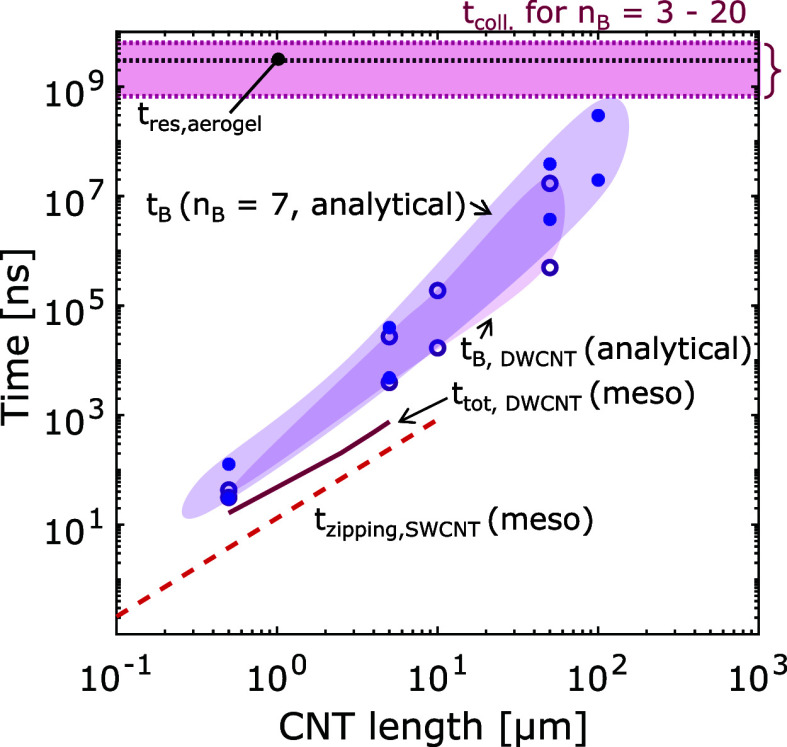
Comparison between reorientation and collision time scales as a function of CNT length. *t*_zipping,SWCNT_ (red dashed line) is the result from mesoscale simulations of between two SWCNTs.^28^ Due to the low initial angle (θ_0_ < θ_c_), only dynamics in the zipping phase was captured. Results from mesoscale simulations used in this study for two DWCNTs (*L* = 0.5–5 μm, θ_0_ = 60°) are shown as the solid line (*t*_tot_ = *t*_B_ + *t*_zipping_). Results from the analytical model for both the reorientation between two DWCNTs (hollow circles) and two *n*_B_ = 7 bundles (filled circles) are shown as shaded areas that enclose the individual cases tested (*L* = 0.5–100 μm, θ_0_ = 60–89°, *E*_rot_ = 0). *t*_coll._ marks the range for the collision time scale in a typical FCCVD reactor for *n*_B_ = 3–20, while *t*_res,aerogel_ ∼ 3 s marks the estimated residence time in the aerogel formation region in an FCCVD reaction.

As mentioned at the beginning of this study, to form an aerogel, the CNTs need to stop forming bundles and start forming a network. The hypothesis was that the onset of an aerogel occurs when the time scale of reorientation *t*_B_ matches the time scale of collisions. Now that the validated semi-analytical model can be applied to describe reorientation between longer CNTs and larger bundles, how does *t*_B_ compare to other process time scales? More importantly, would *t*_B_ match that of collision and hence may lead to aerogel formation? What is a representative time scale for coagulation for an aerosol system consisting of 1D nanoparticles? To answer these questions, the relevant time scales are plotted in Figure 11.

The reorientation time *t*_B_ predicted by the mesoscale and analytical model proposed in this study is plotted as a function of the CNT length in Figure 11. The results from Kateris et al. (red dashed line) were included as *t*_zipping,SWCNT_ although note that due to the limitations with modeling long time scales, only reorientation between SWCNTs with θ_0_ < θ_c_, i.e., zipping only, could be simulated. *t*_tot,DWCNT_ refers to the total reorientation time, i.e., *t*_tot_ = *t*_B_ + *t*_zipping_, and was predicted by the extended mesoscale model in this study for an initial angle of 60° (θ_0_ = 60°). Results from the analytical model for the reorientation between two DWCNTs and two *n*_B_ = 7 bundles are shown as shaded areas that enclose the individual cases tested (*L* = 0.5–100 μm, θ_0_ = 60–89°, *E*_rot_ = 0).

One of the ways to characterize the collision time scale *t*_coll._ for a monodisperse aerosol is the famous formula^29,57^ as shown in eq 9, which denotes the time required for the initial total concentration *N*_tot,0_ to reduce to *N*_tot,0_/*n*_B_
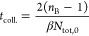
9where β is the coagulation rate coefficient, also known as the collision kernel. The estimated ranges for *t*_coll._ for a typical FCCVD process (β ∼ 0.5 × 10^–13^ m^3^ s^–1^, *N*_tot,0_ ∼ 10^14^ m^–3^^29^) are found to be 0.6 and 6.3 s for *n*_B_ = 3 and 20, respectively, which is a typical range for bundle size from FCCVD processes. This range of time scale is also marked in pink in Figure 11, together with the residence time in the aerogel formation region *t*_res,aerogel_ (∼3 s) for comparison.

It is clear that the predicted range of *t*_B_ by the analytical model (*t*_B_ ∼ 10^–8^–10^–1^ s) hardly overlaps with that of collision (*t*_coll._ ∼ 0.6–6.3 s) calculated via eq 9. This would have suggested no aerogel formation according to the earlier hypothesis, which is contradictory to experimental evidence. Therefore, it is important to re-examine the underlying assumptions in the estimation of the collision time scale and the analytical model. First, the time scale for collision may be different from that proposed byeq 9, which is the time required for the total concentration to decrease by considering the collision kernel β only and ignores any complication caused by particle shape. Second, the proposed analytical model is based on the ideal configuration of two pristine CNTs and does not consider situations such as surface contamination or trapping of catalyst particles on the CNT surface, which may significantly alter the interaction potential. Therefore, the subsequent analysis will propose an alternative method to estimate the collision time scale by considering the 1D shape of CNTs.

Liu et al. proposed that for an aerogel to form from an aerosol system, the system would undergo an “ideal gelation point” (IGP), the point at which the overall effective volume of all clusters has reached that of the system, and the clusters start to “interdigitate”.^26^ This is then followed by the occurrence of a gel that spans the entire volume of the system, also termed as the physical gelation point or the percolation point. This latter phase, which is still an active area of research in aerosol science, features aggregation of both the “sol” cluster and the “gel” cluster and may approach the final gelation state via a different route depending on the cluster concentration in the system.^26^ Nonetheless, the early phase of aerogelation can be reliably described by Brownian dynamics until close to the IGP where the aggregation kinetics may accelerate due to system crowding.

**Figure 12:**
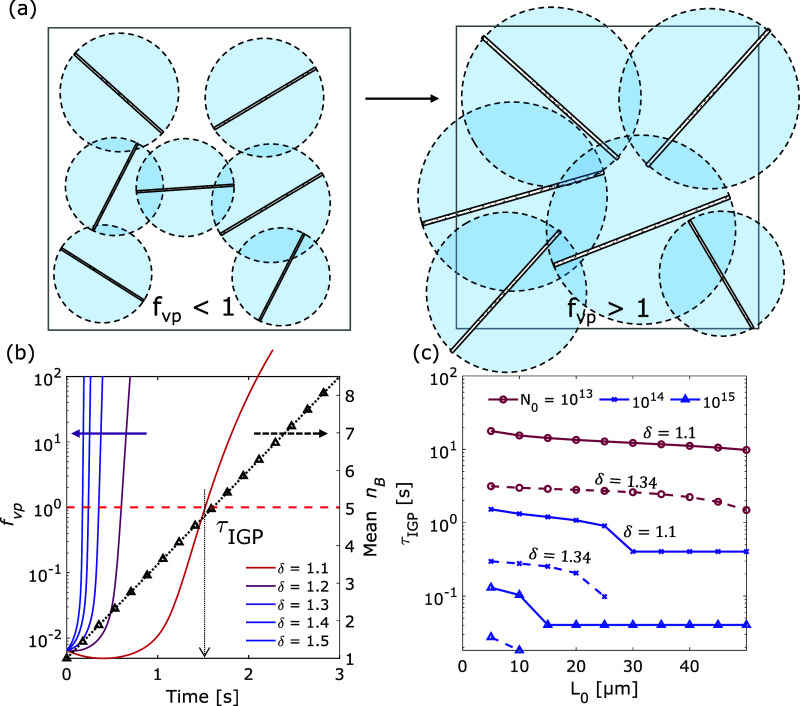
(a) Schematic of how the perimeter volume fraction *f*_vp_ changes as the length of individual particles increases in a 2D projected view. The circles with dashed lines denote the spheres enclosed by the radius of perimeter of the 1D particle. The outer square denotes an arbitrary unit of volume. When *f*_vp_ < 1, the total perimeter volume does not fill the entire volume available. When *f*_vp_ > 1, the total perimeter volume exceeds the entire volume available and particle interdigitating becomes possible. (b) Evolution of the perimeter volume fraction *f*_vp_ and mean bundle size for the elongation ratio ranging from more probable (1.1) to less probable (1.5) with an initial total concentration *N*_tot,0_ = 10^14^ m^–3^. (c) τ_IGP_ calculated for a range of initial total concentrations (1 × 10^13^ to 1 × 10^15^ m^–3^) and lengths (5–50 μm) with β = 0.5 × 10^–13^ m^3^ s^–1^.

Building on this theoretical framework, the time scale was analyzed for the aerogelation of 1D particles. Note that a system of high-aspect-ratio 1D particles is different from a more conventional system consisting of soot particles with a certain fractal dimension (∼2). While the radius of perimeter, i.e., the radius of a sphere that encloses the entirety of the particle, can act as a reasonable measure of size when considering the aggregation dynamics of a soot particle, the radius of perimeter for a 1D cylinder or tube would simply be half of its length, assuming no bending or folding, as shown in Figure 12a. Based on our modeling studies shown here and before,^28^ CNTs quickly form a bundle upon collision, in a manner that mimics coalescence, where statistically, the length of the bundle may elongate by δ as the ends of the CNTs do not necessarily match. Thus, the solid fraction with a CNT cluster/bundle fraction would increase with time as the bundles elongate, while the more conventional system of fractal-like particles, e.g., soot, would not undergo such a step. This difference will affect how the free space between clusters can be evaluated. However, to estimate how long the system takes to reach the IGP, a time-scale analysis can still be carried out by considering how the perimeter volume fraction *f*_vp_ evolves with time and whether it reaches unity within the residence time available in the aerogel formation region in the FCCVD reactor (calculated to be 3–15 s^29^). Here, *f*_vp_ is defined as . We assumed an initially monodisperse CNT length distribution and a constant elongation ratio upon collision δ, i.e., the ratio between the resulting particle length and the initial length, sampled from the Langevin simulation results by Boies et al. Given the rapid reorientation observed earlier, we also assume that reorientation immediately follows every collision event and is completed before the next collision event takes place. Detailed calculations are given in the Supporting Information.

Figure 12b shows how *f*_vp_ changes with time up to 3 s for different values of δ, starting from an initial total concentration *N*_tot,0_ = 1 × 10^14^ m^–3^ and length *L*_0_ of 5 μm. Boies et al. showed that for collisions between CNTs of similar lengths, the collision kernel β would fall within the range of 0.5–1 × 10^–13^ m^3^ s^–1^ for CNT lengths up to 1 mm, so here, a constant collision kernel is adopted at β = 0.5 × 10^–13^ m^3^ s^–1^. Plotted on the right *y*-axis in triangles is the change of the mean bundle size *n*_B_ with time. Although *f*_vp_ is less than 1% initially, it rapidly increases as the bundles lengthen, which with each successive collision approaches unity within *t* = 0.2–1.5 s for elongations of δ = 1.5 and 1.1, respectively. We define the time taken for this value to reach unity as the time scale to reach the IGP, τ_IGP_. The average bundle size can also be determined for IGP at *t* = τ_IGP_, where *n*_B_ ∼ 4.8 and 1.5 for δ = 1.1 and 1.5, respectively.

τ_IGP_ is then estimated for a range of values of *N*_tot,0_ and *L*_0_ as shown in Figure 12c, where the solid and dashed lines denote the results for elongation ratios δ of 1.1 and 1.34, respectively. For high initial concentrations of CNTs, *N*_tot,0_ = 10^15^ m^–3^ (comparable to the catalyst concentration), and average elongation ratio δ = 1.34 (50% of the collision events in terms of cumulative probability), τ_IGP_ is less than 0.1 s, which is significantly shorter than the residence time in an FCCVD reactor. τ_IGP_ is a strong function of the initial length and the elongation ratio δ as *f*_vp_ scales with length to the power three. In an extreme case where the initial length is sufficiently long, the system would start in a post-IGP regime and thus immediately start forming an aerogel. τ_IGP_ also depends strongly on the initial total concentration, whereby a lower initial CNT concentration *N*_tot,0_ would result in a lower initial *f*_vp_ and a slower increase as collision rates scale with the square of particle concentration. For example, initial CNT concentrations of *N*_tot,0_ = 10^14^–10^13^ m^–3^ (indicative of more reasonable catalyst efficiencies of <1%) would result in a τ_IGP_ = ∼ 0.2–2 s (δ = 1.34). Note that ideal gelation is not physical gelation, i.e., the emergence of a gel structure spanning the volume of the reactor. The simulations performed for soot clusters by Liu et al. showed that the time taken for physical gelation is approximately 2 orders of magnitude greater than the time taken to reach IGP. For a 1D-particle system like FCCVD, the relationship between the ideal and physical gelation time scales cannot yet be determined with existing modeling capabilities. However, one can imagine for 1D particles, *f*_vp_ = 1 only marks the start of possible CNTs interdigitating while there remains ample free space within the sphere that encloses each CNT/bundle. Nonetheless, the smaller the τ_IGP_, the more likely an aerogel can eventually form, which favors systems with a greater initial number concentration and length while ruling out operating conditions where τ_IGP_ is too large to be feasible within an FCCVD reactor.

As mentioned at the beginning of this time-scale analysis, although *t*_B_ scales across multiple orders of magnitude depending on the length, the initial collision angle, and the linear density, the range of *t*_B_ barely overlaps with the collision time scale calculated via Friedlander’s formula (0.6–6 s). The collision time scale evaluated by determining the IGP for a system of 1D particles, τ_IGP_, is in the range of 0.2–2 s, which is shorter than that estimated by Friendlander’s formula, but not by orders of magnitude. This means the ranges of *t*_B_ and τ_IGP_ only overlap in the presence of ultralong (>50 μm) CNTs/bundles.

This analysis also opens avenues for future research into probing the later phase of the aggregation dynamics of 1D nanoparticles, i.e., approaching and post-IGP. The monodisperse assumption for length distribution is rather simplistic, while for a polydisperse length distribution, collisions between nanotubes of dissimilar lengths have been shown to result in nearly no elongation,^29^ which would significantly complicate the analysis. Treating the elongation ratio as constant is also likely to result in an inaccurate estimation of the evolution of *f*_vp_. Approaching IGP, Brownian dynamics is likely to fail in describing the aggregation behavior as the system becomes cluster-dense^58^ while the reorientation time scale would continue to rise, approaching that of collision and eventually resulting in the occurrence of a network structure. It is critical to further study phenomena within this process that are not governed by arithmetic means of processes. It is reasonable to hypothesize that the onset of aerogels may be dictated by lower probability events (e.g., CNTs that do not collapse into bundles before additional collision), which nonetheless when occur “nucleate” an aerogel structure, which then spreads through the volume. Therefore, further work must continue to account for statistical variability in processes to examine nucleation phenomena.

## Conclusions

This study investigates the reorientation dynamics between two high-aspect-ratio CNTs by a semi-analytical model based on a second-order ODE, the results from which are in agreement (<%10 in terms of reorientation time) with those from mesoscale simulations but incur minimal computational cost (<0.1% compared to equivalent cases in the mesoscale model). High-aspect-ratio (up to ∼10^4^) CNTs and their bundles are typical of products from an FCCVD process. Their reorientation behavior spans across multiple orders of magnitude in length scale and time scale and hence is too challenging for current atomistic or mesoscale MD simulation approaches but can be captured by the proposed semi-analytical model. A simplified method for estimating the “critical angle”, the point where reorientation transits from “central rotation” into the “zipping” regime, is also proposed and used as the stopping condition for the analytical model. The estimated critical angles agree well with those found by the mesoscopic models (error ∼2°). The proposed analytical model allows for the incorporation and scaling of various physical phenomena in a flexible manner. Therefore, it not only facilitates our understanding of the interplay between various physical effects but can also be adapted to suit other 1D-particle systems as well.

The model reveals that the reorientation time is, in descending order, dictated by the CNT length, the initial collision angle, and the linear density, such that the resulting reorientation time can span across several orders of magnitude from a few nanoseconds to ∼0.1 s. It is also affected by the initial rotational energy and drag, the latter of which is often ignored in atomistic or even mesoscopic simulations due to high computational cost but was shown to potentially lengthen the reorientation process by up to 2 orders of magnitude. The scaling factors incorporated in the model further allow for the description of bundle–bundle reorientation. Counterintuitively, bundle–bundle reorientation is not always slower than that between individual tubes, as the driving force (i.e., the torque due to vdW interaction) is sensitive to the contact configuration. Thus, although CNT bundles have a higher second moment of inertia, the torque increases in discrete steps as the bundle size increases and can result in faster reorientation in certain scenarios, e.g., from *n*_B_ = 1 to *n*_B_ = 2. Therefore, the reorientation time scale is obtained for CNTs/bundles with a wide range of parameters that *t*_B_ = 10 ns to 0.1 s for a CNT length of 0.5–100 μm. This was then compared to the collision time scale, which was approximated as the time required to reach IGP for a system governed by Brownian dynamics with initial conditions representative of a typical FCCVD reactor (τ_IGP_ = 0.2–2 s). The time-scale analysis shows that IGP can be reached within the residence time available in the reactor (∼3 s) if the process starts with long CNT lengths and/or large number concentrations (>10^14^ m^–3^). However, the collision time scale is always greater than reorientation time scales until the emergence of ultralong and large bundles, which is challenging to achieve in typical synthesis conditions. It is hence important to further study physical phenomena that deviate from the ideal configuration assumed in studies so far while accounting for the statistical variability within this unique gas-phase process.

Overall, the proposed semi-analytical model, with low computational cost and reasonable accuracy, offers insights into the various physical phenomena during the reorientation process of high-aspect-ratio CNTs and their bundles. The results enable bundle formation, a unique aerosol phenomenon in CNT synthesis from FCCVD reactors, to be considered alongside network and eventual aerogel formation. Moreover, the semi-analytical model can be extended to study other systems consisting of high-aspect-ratio 1D nanoparticles and hence open new research avenues into understanding the gas-phase self-assembly of advanced 1D nanomaterials.

## Supporting Information Available

Detailed descriptions of the methods for calculating the stretching and bending force constants, torque due to drag, the critical angle, and the IGP time scale; additional results on the validation of the MWCNT mesoscale model against atomistic simulation, and bundle–bundle reorientation (PDF)
