# Supplementary material for: Gas-Phase Dynamics of Bundle Formation from High-Aspect-Ratio Carbon Nanotubes
Source: Langmuir. 2024 Sep 30;40(41):21460–75. doi: 10.1021/acs.langmuir.4c02260 (PMC11483740; doi:10.1021/acs.langmuir.4c02260)
Supplement: Supplementary file 1 — la4c02260_si_001.pdf [file la4c02260_si_001.pdf]

Supporting Information for Gas-phase dynamics of bundle formation from high-aspect-ratio carbon nanotubes

Rulan Qiao,[†] Xiaoyu Qiu,[†] and Adam Boies[*,†,‡]

†*Department of Engineering, University of Cambridge, Cambridge, United Kingdom.*

‡*Department of Mechanical Engineering, Stanford University, Stanford, CA 94305, United States.*

E-mail: amb233@cam.ac.uk

Phone: +44 1223 746972. Fax: +44 1223 765 311

This document contains 12 pages, 5 figures, and 13 equations.

Table of contents

1 Evaluation of force constants **S2**

2 Drag due to interaction with gas media **S5**

3 Estimation of the critical angle **S6**

4 Validation of MWCNT mesoscale model against atomistic simulations **S7**

5 Bundle-bundle reorientation **S9**

6 The timescale for reaching ideal gelation point (IGP) **S10**

1 Evaluation of force constants

When calculating bending force constants, an issue arises due to the tilted top surface as shown in Figure S1a. Curvature at the maximum position can be acquired either from the slope of this top surface or the position of its centre. However, the top surface deviated from the ideal shape during bending (Figure S1a right), which causes the calculated curvature to be lower than the curvature corresponding to that energy, as depicted in S1b. However, it is not feasible to directly use the curvature from the ideal shape, which means the curvature is evaluated from the displacement of the position of the CNT top centre (Figure S1a left). This ideal curvature overestimates the extent of bending, leading to an underestimated k_{bnd} as shown in Figure S1c. Thus, we employ an integration method based on the assumption that the curvature changes linearly from the higher perfect curvature at the bottom, κ_{bottom}, to the lower tilted curvature at the top, κ_{top}, $\kappa_{\mathrm{bottom}} > \kappa_{\mathrm{top}}$. The bending strain energy, E_{bnd}, is given by,

$$E_{\mathrm{bnd}} = \frac{1}{2} k_{\mathrm{bnd}} L_{\mathrm{CNT}} \kappa^2 \tag{1}$$

where L_{CNT} is the length of CNT and κ is the corresponding curvature, $\kappa = 1/R_{\mathrm{curv}}$, with R_{curv} is radius of curvature. $1/2$ k_{bnd} is estimated from the gradient in the plot of $E_{\mathrm{bnd}}/L_{\mathrm{CNT}}$ against κ^2. The theoretical gradient of this line is $1/2 \times k_{\mathrm{bnd}} = 10400$ eV/Å.[1,2] As an illustrative example, we conducted this dynamic simulation of a 5 nm (10,10) SWCNT, repeating it 26 times with various forces. k_{bnd} calculated from the flatter κ_{top} (Figure S1b), 35640 eV Å, is much higher than the theoretical value of 20800 eV Å, whereas k_{bnd} calculated from the ideal κ_{bottom} (Figure S1c), 13284 eV Å, is much lower. Thus, an integration method is applied to deal with this inaccurate curvature. Curvature of an infinitesimal nanotube segment with a distance L_{curv} away from the bottom is determined by the linear assumption,

$$\kappa(L_{\mathrm{curv}}) = \kappa_{\mathrm{bottom}} + (\kappa_{\mathrm{top}} - \kappa_{\mathrm{bottom}}) \frac{L_{\mathrm{curv}}}{L_{\mathrm{CNT}}}. \tag{2}$$

The strain energy of nanotube with changing curvature is calculated from an integration,

$$E_{\text{bnd}} = \int_0^{L_{\text{CNT}}} \frac{1}{2} k_{\text{bnd}} \kappa(L_{\text{curv}})^2 \, \mathrm{d}L_{\text{curv}}. \tag{3}$$

Substituting eq 2 into eq 3 and integrating, E_{bnd} gives

$$E_{\text{bnd}} = \frac{1}{2} k_{\text{bnd}} L_{\text{CNT}} [\kappa_{\text{bottom}}^2 + \kappa_{\text{bottom}}(\kappa_{\text{top}} - \kappa_{\text{bottom}}) + \frac{(\kappa_{\text{top}} - \kappa_{\text{bottom}})^2}{3}]. \tag{4}$$

The term in the square brackets is equivalent to a corrected curvature, $\kappa_{\text{corrected}}$, of this slightly deformed structure. Therefore, an accurate k_{bnd} can be evaluated as usual from this corrected curvature as shown in Figure S1d. k_{bnd} calculated from the dynamic simulation of 5 nm CNT is highly consistent with the theoretical value, for example $k_{\text{bnd,MD}} = 20100$ eV Å versus $k_{\text{bnd,Theory}} = 20800$ eV Å.

The bending simulation is isotropic in all the directions for a cylindrical nanotube. However, it is possible for the axial simulation to be asymmetric in the compressive direction and the tensile direction. We determine the stretching force constants attained from the simulations are different under compressive and tensile forces as depicted in Figure S2a and Figure S2b, respectively. k_{str} is defined by,

$$E_{\text{str}} = \frac{1}{2} k_{\text{str}} L_{\text{CNT}} {\epsilon_{\text{str}}}^2 \tag{5}$$

where ϵ_{str} is the stretching strain, $\epsilon_{\text{str}} = \Delta L / L_{\text{CNT}}$, and ΔL is the change in nanotube length under axial deformation. k_{str} is evaluated from the gradient in the graph of $E_{\text{str}}/L_{\text{CNT}}$ against ϵ_{str}^2. As depicted in Figure S1 and S2, a constant offset is observed, which represents the thermal fluctuation energy without any applied strain. This offset corresponds to the thermal energy associated with one degree of freedom, calculated as 1/2 kT. At a temperature, the thermal energy is approximately 0.0043 eV ($1/2 \times 1.38 \times 10^{-23}$ [J/K]/(1.6×10^{-19} [J/eV]) $\times$ 100 K= 0.0043 eV). The y-axis intercept is about 0.0001 eV/Å. For a 5 nm CNT, this

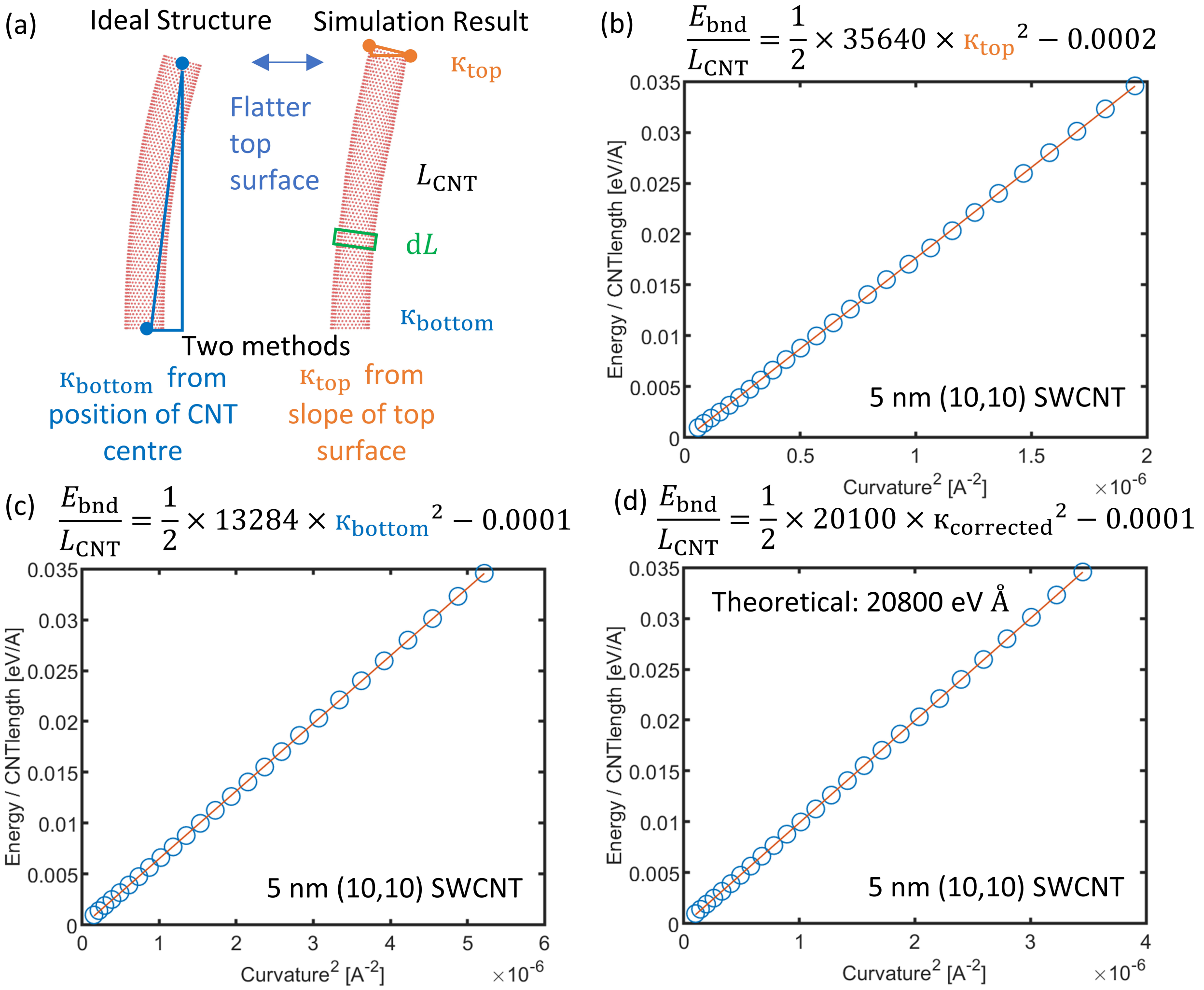

Figure S1: (a) Schematic of the deformation issue arising from the tilted top surface. (b) Overestimated bending force constant (35640 eV Å in the equation compared with the theoretical value, 20800 eV Å) calculated from the slope of top surface. (c) Underestimated bending force constant (13284 eV Å) calculated from the position of CNT centre. (d) Accurate bending force constant (20100 eV Å) calculated from the corrected curvature.

offset is about 0.005 eV. This value is consistent with the expected scale of thermal energy, supporting its physical relevance in the model. Perfect linear fitting is observed for the repeated simulations. However, the compressive stretching force constant is slightly higher than the tensile force constant. It is consistent with the inspection by Brenner[3] that the short-range REBO bond energy rises more rapidly under compression than under tension. The stress-strain curves also behave differently in Natsuki's continuum shell model of CNT.[4] For the mechanical propertise of CNTs, it is commonly to use the tensile force constant

instead of the compressive force constant. There is a good agreement between the tensile force constant obtained from linear fitting, 749 eV/Å, and the theoretical result calculated from the equation by Zhigilei et al., 768 eV/Å.[2] Thus, we only take the tensile stretching force constant into account in our simulations.

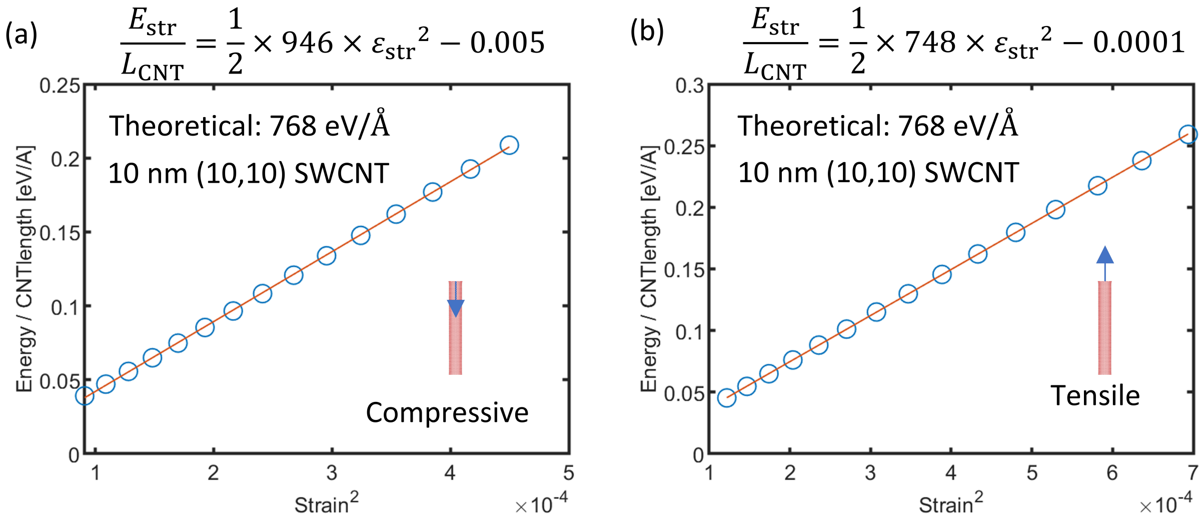

Figure S2: Stretching force constants obtained from (a) compressive forces (946 eV/Å), and (b) tensile forces (748 eV/Å).

2 Drag due to interaction with gas media

The torque due to drag is derived from eq 3 as follows. The two limiting values of momentum accommodation factor φ are 0 and 1, for specular and diffuse scattering, respectively. We assume $\varphi = 0.9$, following the recommendation by Millikan based on experimental data. β is the orientation of the body with respect to the drift velocity such that $\beta = \pi/2$ if the cylinder is perpendicular to the drift velocity. If both CNT molecules are rotating around central positions, only the perpendicular component of drag affects the reorientation dynamics, i.e. $\beta = \pi/2$. The torque due to drag can be integrated along the CNT axis, which gives $T_{\text{drag}} = \frac{1}{24} c_{\text{D}} L^3 \cdot \dot{\theta}$

$$T_{\text{drag}} = \int_{x=-L/2}^{x=L/2} dT_{\text{drag}} = \int_{x=-L/2}^{x=L/2} x \cdot F_{\text{D}}(x)dx = \int_{x=-L/2}^{x=L/2} c_{\text{D}} x^2 (\frac{\dot{\theta}}{2})dx = \frac{1}{24} c_{\text{D}} L^3 \cdot \dot{\theta}, \quad (6)$$

where the drag coefficient per unit length $c_D = \sqrt{2\pi m_g kT}\left[\varphi + (2 - \frac{6-\pi}{4}\varphi)\sin^2\beta\right] NR$. Within the model, this is further incorporated into c_1 as $\frac{L^3}{24}c_D(\beta = \frac{\pi}{2})$ so that $T_{\text{drag}} = c_1 \cdot \dot{\theta}$.

3 Estimation of the critical angle

We proposed a method to estimate the critical angle by matching the rate of change in intermolecular energy with that in bending energy. Rearranging eq 7 gives

$$\cot\theta \cdot \csc^2\theta \cdot U_{\text{vdW}}^2(\theta) \cdot \frac{L'}{2k} = \left(\frac{L'}{2k}\cot^2\theta \cdot U_{\text{vdW}}(\theta) + 1\right) \cdot \frac{\partial U_{\text{vdW}}}{\partial\theta}. \tag{7}$$

The estimated values are compared to those obtained from mesoscale simulations in Figure S3. The agreement is found to be acceptable, with the analytical estimation under-predicting the critical angle by ∼2° for all the three lengths tested. This discrepancy in the stopping condition in the analytical model would lead to the a 1-4% difference in the predicted reorientation time, which is not significant for the aim of this study. A small discrepancy is expected since the relative angle in mesoscale simulations is calculated based on the position of the end segments of the CNT molecules, which, in case of a small amount of bending, would be slightly larger than the local relative angle at the centre of the two CNTs. Therefore, the proposed method was sufficient to estimate the critical angles for reorientation between longer CNTs (L >5 µm). The results show that θ_c is a strong function of CNT length, such that when the CNT length reaches 5 µm, the critical angle would be more than 85°. Here a maximum cut-off of 85° is set for θ_c, because the simplified method, based on the rate of change of intermolecular energy, would become inaccurate and not physically meaningful beyond such high relative angle. Nonetheless, the trend still holds and Figure S3 can be used as a guide to determine whether the reorientation dynamics would be dominated by time spent in central rotation. For example, two 2.5 µm DWCNTs with an initial relative angle of around 80° would spend a long time undergoing central rotation until the relative angle reduces to 25°, when the reorientation would transition into

the 'zipping' regime and proceed rapidly. However, two CNTs that are tens of microns in length would enter the 'zipping' regime directly, resulting in much more rapid reorientation time than expected. Therefore, it is important to have a reasonable estimation of the critical angle in order bound the analytical model within its valid region.

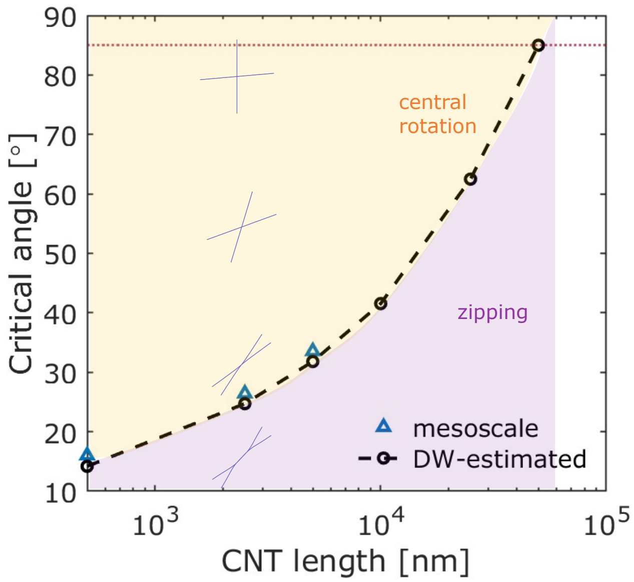

Figure S3: Comparison between the mesoscale simulation and the analytical estimation of the critical angle θ_c for two DWCNTs with various length.

4 Validation of MWCNT mesoscale model against atomistic simulations

The mesoscale model of MWCNTs we utilized for bundle simulations is validated by comparison with atomistic simulations. In Figure S4, the solid lines and dashed lines represent the energy change in atomistic models and mesoscale models, respectively. The simulations of (10,10) double-walled carbon nanotubes (DWCNTs) at different collision angles, 15° (blue), 20° (red) and 30° (yellow), are shown in Figure S4a. DWCNTs with different chirality, (10,10) (yellow) and (30,30) (purple), at 30° are shown in Figure S4c. All the intermolecular energies are normalized by the mean energy after reorientation, ratio = (mean – energy)/mean, to emphasize the comparison in time rather than differences in energy as depicted in Figure

S4b and S4d.

The energy changes of the same nanotube rise with relative angles (Figure S4a), due to the decrease in interaction area, resulting in higher initial intermolecular energies. The energy released in atomistic model is higher than the energy in mesoscale model. We speculate that the atomistic CNTs have a larger contact area due to partial flattening on the surface. The mesoscopic CNT is assumed to constitute perfect cylindrical segments that do not deform. Thus, the energy discrepancy from vdW attraction in atomistic model is higher due to this deformation. This also results in a stronger attraction between CNTs in atomistic simulations. The reorientation time in the atomistic model is hence shorter than that in the mesoscale model, as depicted in Figure S4. This discrepancy in energy and reorientation time is larger for (20,20) DWCNTs (Figure S4c) than (10,10) DWCNTs, due to the larger contact area affected by local deformation. This is also consistent with the rapid bundling in atomistic model (Figure S4d). Good agreement is generally found between the mesoscale and the atomistic models after accounting for the decrease in bundling time in atomistic model due to flattening. Thus, the optimized MWCNT mesoscale model is used for reorientation simulations.

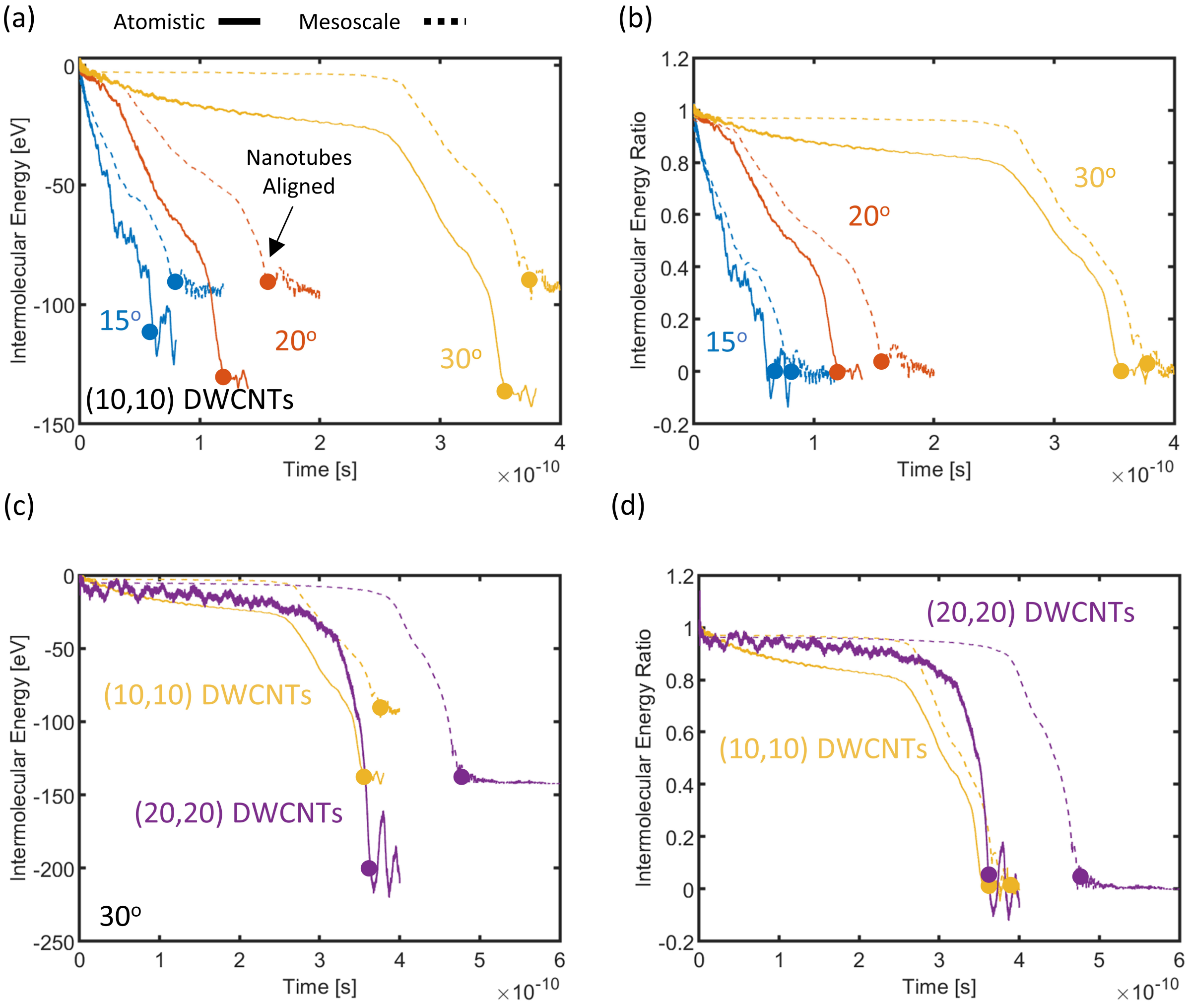

Figure S4: (a) Comparison between mesoscale simulation results and atomistic simulation results of (10,10) DWCNTs at different collision angles, 15° (blue), 20° (red), and 30° (yellow). The dots on the plot represent the time when realignment finishes. (b) Normalized results of (a) by the mean energy after reorientation to emphasize the time comparison. (c) Comparison between mesoscale simulation results and atomistic simulation results of (10,10) DWCNTs and (20,20) DWCNTs both at 30°. (d) Normalized results of (b) by the mean energy after reorientation.

5 Bundle-bundle reorientation

Figure S5 shows the reorientation between two 0.5 µm CNT bundles with a size from $n_{\mathrm{B}} = 1$ to 7. For $n_{\mathrm{B}} > 1$, it is assumed that the vdW interaction is scaled by four times ($k_{\mathrm{vdW}} = 4$), i.e. no complication such as internal twisting within each bundles.

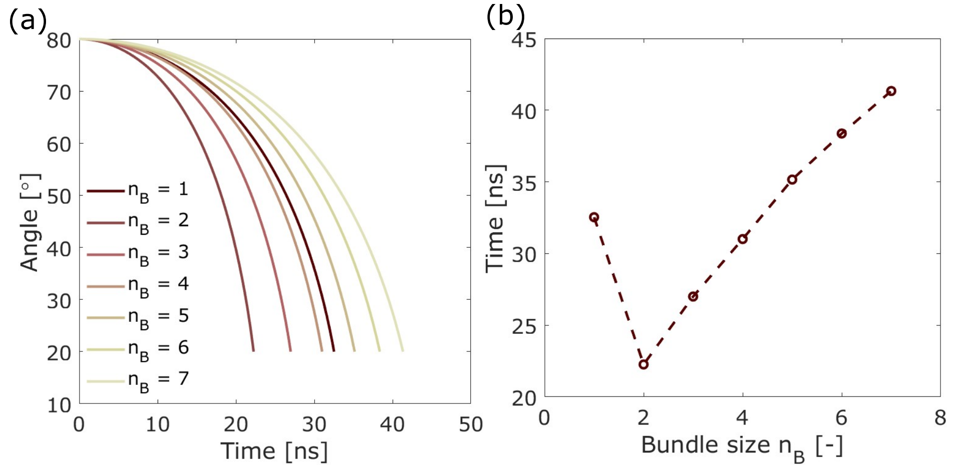

Figure S5: Reorientation between two 0.5 µm CNTs/bundles with a bundle size from $n_{\mathrm{B}} = 1$ (individual CNTs) to $n_{\mathrm{B}} = 7$ shown as (a) the evolution of relative angle with time and (b) the predicted reorientation time t_{B} as a function of bundle size.

6 The timescale for reaching ideal gelation point (IGP)

Collisions within an initially monodisperse aerosol system that follows Brownian dynamics can be described as following.[6]

$$N_{\mathrm{tot}}(t) = \frac{N_{\mathrm{tot},0}}{1 + t/\tau} \tag{8}$$

where N_{tot} denotes the total number concentration and τ_{B} is the characteristic time, i.e. the time at which the total number concentration would halve, which is a function of the initial number concentration and the collision kernel β (assumed to be constant) and can be described as:

$$\tau_{\mathrm{B}} = \frac{2}{\beta N_{\mathrm{tot},0}} \tag{9}$$

Furthermore, the number concentration of particles of bundle size n_{B} can be written as:

$$N_{n_{\mathrm{B}}}(t) = N_{\mathrm{tot},0} \cdot \frac{(t/\tau)^{n_{\mathrm{B}}-1}}{(1 + t/\tau)^{n_{\mathrm{B}}+1}} \tag{10}$$

while the length of particles of bundle size n_B is:

$$L_{n_\mathrm{B}} = L_0 \cdot \delta^{n_\mathrm{B}-1} \tag{11}$$

Therefore, one can evaluate the total perimeter volume fraction f_vp at any given time. Here we regard the point at which this value reaches unity to be the IGP.

$$f_\mathrm{vp} = \frac{\pi}{6} \cdot \sum_{n_\mathrm{B}=1}^{\infty} N_{n_\mathrm{B}} \cdot L_{n_\mathrm{B}}^3 \tag{12}$$

One can also evaluate the average bundle size by

$$\bar{n_\mathrm{B}} = \frac{\sum_{n_\mathrm{B}=1}^{\infty} N_{n_\mathrm{B}}(t) \cdot n_\mathrm{B}}{N_\mathrm{tot}(t)} \tag{13}$$

In summary, assuming a monodisperse initial length distribution of particles with a total number concentration $N_\mathrm{tot,0}$ and length L_0, a constant collision kernel β, and a constant elongation ratio δ, the evolution of the total perimeter volume fraction f_vp for a system undergoing Brownian collisions can be determined and hence the time taken to reach IGP can be estimated.

References

(1) Vaccarini, L.; Goze, C.; Henrard, L.; Hernandez, E.; Bernier, P.; Rubio, A. Mechanical and electronic properties of carbon and boron-nitride nanotubes. *Carbon* **2000**, *38*, 1681–1690.

(2) Zhigilei, L. V.; Wei, C.; Srivastava, D. Mesoscopic model for dynamic simulations of carbon nanotubes. *Phys. Rev. B* **2005**, *71*, 165417.

(3) Brenner, D. W.; Shenderova, O. A.; Harrison, J. A.; Stuart, S. J.; Ni, B.; Sinnott, S. B.

A second-generation reactive empirical bond order (REBO) potential energy expression for hydrocarbons. *J. Phys.: Condens.Matter* **2002**, *14*, 783–802.

(4) Natsuki, T.; Endo, M. Stress simulation of carbon nanotubes in tension and compression. *Carbon* **2004**, *42*, 2147–2151.

(5) Millikan, R. A. The general law of fall of a small spherical body through a gas, and its bearing upon the nature of molecular reflection from surfaces. *Phys. Rev.* **1923**, *22*, 1–23.

(6) Friedlander, S. K. *Smoke, dust, and haze: fundamentals of aerosol dynamics*, 2nd ed.; New York, Oxford University Press, 2000.
